# Role of Lipids in Spheroidal High Density Lipoproteins

**DOI:** 10.1371/journal.pcbi.1000964

**Published:** 2010-10-28

**Authors:** Timo Vuorela, Andrea Catte, Perttu S. Niemelä, Anette Hall, Marja T. Hyvönen, Siewert-Jan Marrink, Mikko Karttunen, Ilpo Vattulainen

**Affiliations:** 1 Department of Physics, Tampere University of Technology, Tampere, Finland; 2 Department of Medicine and Center for Computational and Structural Biology, University of Alabama, Birmingham, Alabama, United States of America; 3 VTT Technical Research Centre of Finland, Espoo, Finland; 4 Department of Physics, University of Oulu, Oulu, Finland; 5 Groningen Biomolecular Sciences and Biotechnology Institute and Zernike Institute for Advanced Materials, University of Groningen, Groningen, The Netherlands; 6 Department of Applied Mathematics, The University of Western Ontario, London, Ontario, Canada; 7 Department of Applied Physics, Aalto University School of Science and Technology, Espoo, Finland; 8 MEMPHYS–Center of Biomembrane Physics, Physics Department, University of Southern Denmark, Odense M, Denmark; Max Planck Institute for Biophysical Chemistry Göttingen, Germany

## Abstract

We study the structure and dynamics of spherical high density lipoprotein (HDL) particles through coarse-grained multi-microsecond molecular dynamics simulations. We simulate both a lipid droplet without the apolipoprotein A-I (apoA-I) and the full HDL particle including two apoA-I molecules surrounding the lipid compartment. The present models are the first ones among computational studies where the size and lipid composition of HDL are realistic, corresponding to human serum HDL. We focus on the role of lipids in HDL structure and dynamics. Particular attention is paid to the assembly of lipids and the influence of lipid-protein interactions on HDL properties. We find that the properties of lipids depend significantly on their location in the particle (core, intermediate region, surface). Unlike the hydrophobic core, the intermediate and surface regions are characterized by prominent conformational lipid order. Yet, not only the conformations but also the dynamics of lipids are found to be distinctly different in the different regions of HDL, highlighting the importance of dynamics in considering the functionalization of HDL. The structure of the lipid droplet close to the HDL-water interface is altered by the presence of apoA-Is, with most prominent changes being observed for cholesterol and polar lipids. For cholesterol, slow trafficking between the surface layer and the regimes underneath is observed. The lipid-protein interactions are strongest for cholesterol, in particular its interaction with hydrophobic residues of apoA-I. Our results reveal that not only hydrophobicity but also conformational entropy of the molecules are the driving forces in the formation of HDL structure. The results provide the first detailed structural model for HDL and its dynamics with and without apoA-I, and indicate how the interplay and competition between entropy and detailed interactions may be used in nanoparticle and drug design through self-assembly.

## Author Summary

Cardiovascular diseases are the primary cause of death in western countries. One of the main causes is lipid accumulation and plaque formation on arterial walls, called atherosclerosis. The risk of being exposed to this condition is reduced by high levels of high density lipoprotein (HDL). The functionality of HDL has remained elusive, and even its structure is not well understood. Through extensive coarse-grained simulations, we have clarified the structure of the lipid droplet in HDL and elucidated its interactions with the apolipoprotein A-I (apoA-I) that surrounds the droplet. We have found that the structural and dynamic properties of lipids depend significantly on their location in the particle (core, intermediate region, surface). As for apoA-I, we have observed it alter the overall structure of the lipid droplet close to the HDL-water interface, with prominent changes taking place for cholesterol and other polar lipids. The nature of lipid-protein interactions is most favorable for cholesterol. Our results reveal that not only hydrophobicity but also conformational entropy are the driving forces in the formation of HDL structure, suggesting how the interplay and competition between entropy and detailed interactions may be used in nanoparticle and drug design through self-assembly.

## Introduction

Cardiovascular diseases are the primary cause of death in western countries [1]. One of the main causes is the lipid accumulation and plaque formation on arterial walls, called atherosclerosis. This eventually leads to the narrowing of arteries, plaque rupture, clotting, and potential death. Generally speaking, high levels of low density lipoprotein (LDL) in blood have been found to increase the risk of atherosclerosis [2], [3], whereas high levels of high density lipoprotein (HDL) have been shown to reduce the risk [4], [5].

Despite more than a decade of extensive studies, LDL and HDL structures are not well understood. This is largely due to their small size which ranges from about 10 (HDL) to 25 nm (LDL) rendering experimental studies of the detailed lipoprotein structures extremely difficult. This challenge is further corroborated by the soft nature of lipoparticles whose structures are transient due to thermal forces driving molecular assembly processes in living matter. The challenge is to unravel the role and mechanisms of lipoproteins in the trafficking of cholesterol and in the cholesterol-based diseases. In this work, we focus on HDL.

Let us briefly summarize the main insight one has about high density lipopproteins. HDL particles are comprised of a lipid droplet surrounded by proteins [6], [7]. Apolipoprotein A-I (apoA-I) is the main protein associated with HDL, which is the main carrier of excess cholesterol from peripheral tissues to the liver, that is, for reverse cholesterol transport [5], [8]. After synthesization, the ATP-binding cassette transporter A1 (ABCA1) assembles lipid-poor apoA-I molecules and lipids into discoidal HDL particles [9], after which the enzyme lecithin∶cholesterolacyl transferase (LCAT) esterifies cholesterol molecules, leading to the formation of spheroidal HDL [10], [11]. The spheroidal HDL is the main form of HDL responsible for cholesterol transport to the liver.

Though a number of experimental studies have been carried out to unravel the structure and dynamics of apoA-I molecules in lipid-free form [12]–[15] and in discoidal HDL complexes [16], [17], the structure of the spheroidal HDL has remained unclear. As for the structure of apoA-I, a large amount of data is in favor of the so-called double belt model (see Ref. [18] and references therein), where the apoA-I proteins line along the lipid droplet. The composition of the droplet has been resolved [19], [20] (see Table 1), indicating free cholesterol (CHOL), cholesteryl esters (CE), triglycerides (TG), phospholipids, and lysolipids to be its main constituents, distributed such that there is a hydrophobic interior of triglycerides and cholesteryl esters and a surface covered by polar head groups of phospholipids [21]. This is essentially the so-called *two-layer model* for HDL [6], [7], [22]. Furthermore, parts of the apoA-I proteins have been proposed to interact with the acyl chains of the lipids [23]–[27].

**Table 1:** Molecular composition of HDL.

Component	N (simulation)	mol % (simulation)	mol % (experiment)
POPC	260	53.9	}56.1
PPC	10	2.1	
CE	122	25.3	25.5
CHOL	49	10.2	10.2
TG	39	8.1	8.2
ApoA-I	2		

The table shows the number of molecules (N) used in the simulations, together with the experimentally measured molar percentages in human plasma HDL [19]. While this choice reflects the relative amounts observed for the different molecular components in HDL, there is reason to stress that HDL composition is very heterogeneous and depends on, e.g., HDL size and diet [66], [67]. The present study essentially represents the largest HDLs, that is 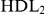; the effect of changes in lipid and protein concentrations are discussed below in this article. The abbreviations: POPC (palmitoyl-oleoyl-phosphatidylcholine), PPC (palmitoyl-PC), CE (cholesteryl ester, here cholesteryl oleate), CHOL (free cholesterol), TG (triglyceride, here trioleate).

Currently, the role of lipids for HDL functions are only vaguely understood. This is partly due to the transient time scales associated with and the nano-scale nature of HDL. Further issues include the poor understanding of lipid organization and interplay of lipids with apoA-I. Considering findings that lipids are an integral component of protein structures, e.g., in membrane proteins that are in constant interplay with lipids [28], it is obvious that clarifying the role of lipids for HDL properties is extremely important.

A number of computational studies have recently been conducted to complement experiments. Previous computational studies of HDL particles have focused on discoidal particles consisting of phospholipids and two apoA-I molecules [29]–[32]. These studies have provided some insight into the mechanisms of assembly and the dependence of the particle shape on the lipid/protein molar fractions. In a different approach, bulk melts of cholesteryl esters [33] and triglycerides [34], as well as combination of cholesteryl esters with POPCs [35] have recently been simulated. Catte *et al.* [36] reported the first computational approach towards understanding the structure of spheroidal HDL particles. Their study clarified the conformation of apoA-I in model spheroidal HDL particles using both all-atom (AA) and coarse-grained (CG) molecular dynamics (MD) simulations. This combination of AA and CG-MD simulations led to model spheroidal HDL particles with prolate ellipsoidal shapes having sizes consistent with experimental results and suggested that cholesteryl esters stabilize the conformations of apoA-I [37]. In a more recent work, Shih et al. also combined coarse-grained simulations with atomistic ones in a series of simulations where discoidal HDL was matured into spherical HDL upon incorporation of cholesteryl esters [38]. They found that maturation results from the formation of a dynamic hydrophobic core composed of cholesteryl esters, the core being surrounded by a layer of phospholipids and apoA-I proteins. Interestingly, Shih et al. also fine-grained the coarse-grained HDL particle to atomistic description and then used atomistic simulations to consider the structure of apolipoproteins around the lipid droplet, and in particular the importance of salt bridges in apoA-I.

The main limitations of previous simulations of HDL particles are two-fold. First, the lipid composition modeled in recent simulations has been somewhat unrealistic: instead of a many-component lipid mixture, the lipid droplet has been modeled as a single-component POPC melt, or as a mixture of POPCs and cholesteryl esters [36]. The role of the many different lipid species in HDL has therefore remained unknown. Second, the time scales of HDL simulations have been too short compared to the characteristic time scales of lipid mixing and structural deformations associated with HDL. As even the time scale of lipid mixing is of the order of 1 
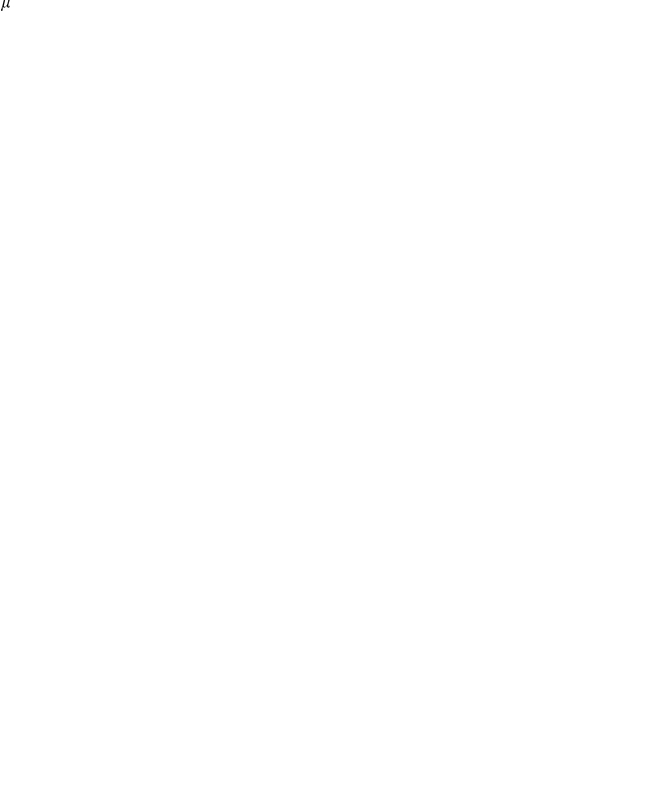
s (derived through the diffusion of lipids inside HDL), and the current state-of-the-art for atomistic simulations of HDL extends over 10–100 ns, it seems obvious that currently atomistic simulations are not the method of choice for dealing with HDL over large enough time scales.

**Figure 1:**
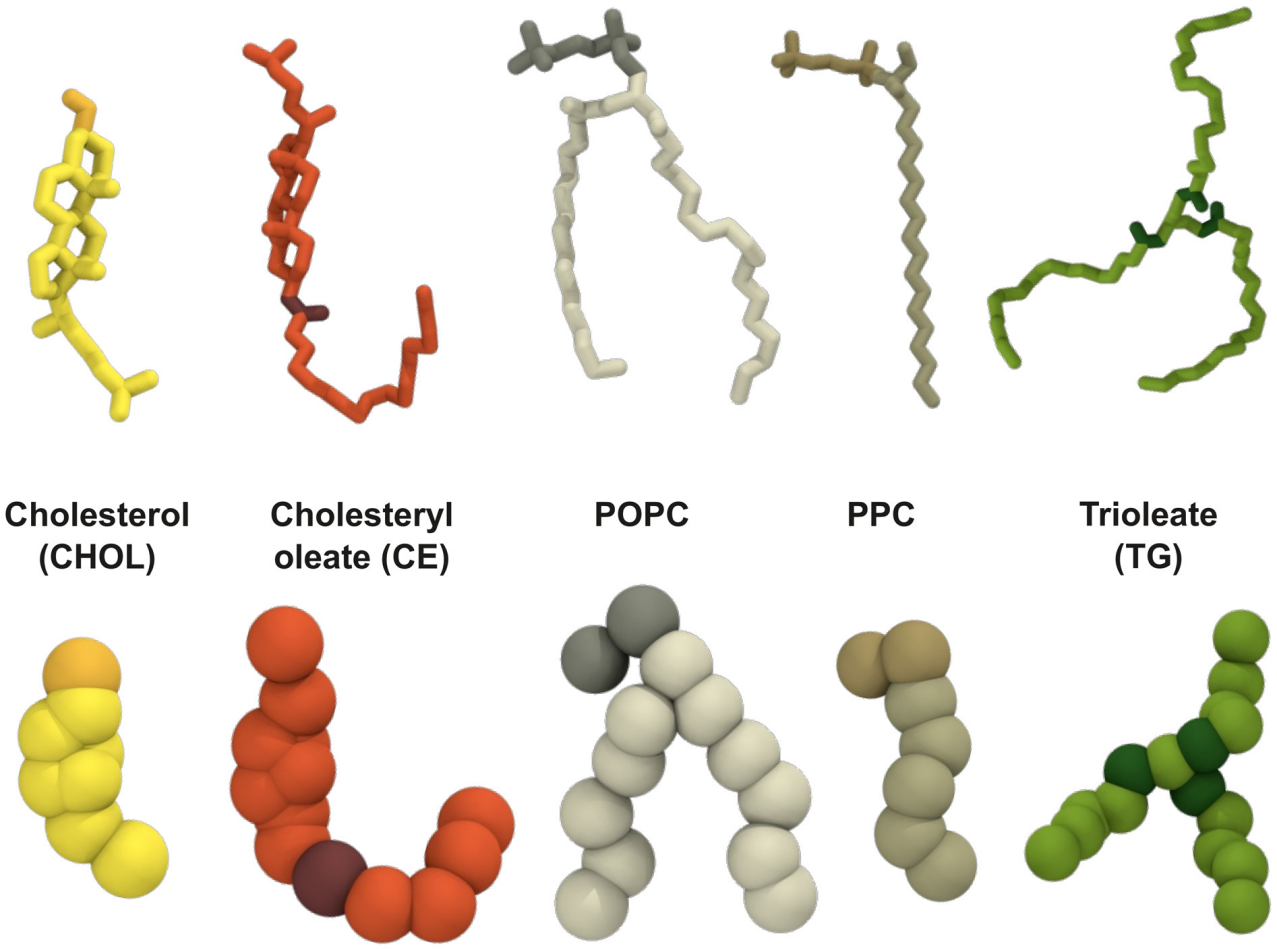
Descriptions of the molecules considered in the study. (Top) Atomistic (united atom) descriptions, and (bottom) the coarse-grained representations.

**Figure 2:**
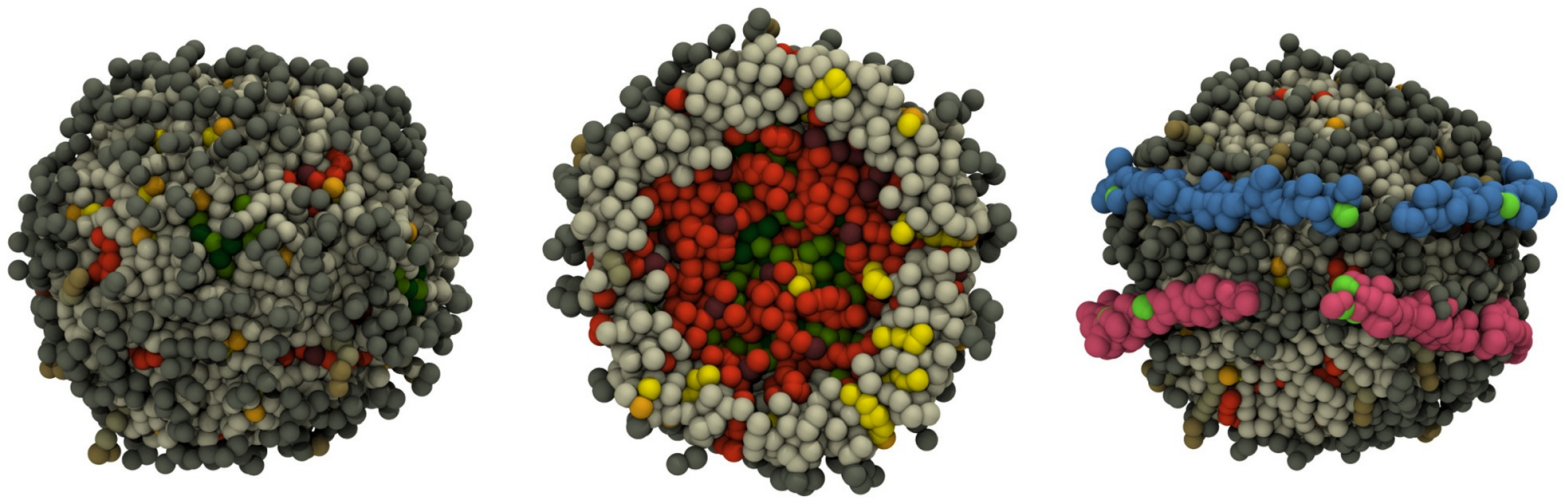
Example of a protein-free lipid droplet (left), its molecular distribution shown through a slice across the particle (middle), and HDL including two apoA-I proteins (right). Dark gray stands for POPC headgroup and dark brown for PPC headgroups, light gray for POPC hydrocarbon chains, light brown for PPC chains, light orange for CHOL OH-groups, bright yellow for cholesterol body, dark orange for CE ester bond, orange for CE ester body and chain, dark green for TG ester bonds, and bright green for TG chain. In HDL, proline residues in apoA-I sequences are in green.

Our objective is to overcome the above limitations. We have performed MD simulations of spheroidal HDL particles using the full lipid composition of human plasma HDL [19]. Instead of atom-scale simulations, we employ the coarse-grained MARTINI model [39], [40] that has performed exceptionally well in a number of studies dealing with lipids and proteins [36], [39]–[42]. We consider both the protein-free lipid droplets and the full HDL particles containing also two apoA-I molecules around the droplet, see Figures 1 and 2. Composition of the HDL system is described in Table 1 with abbreviations of all molecules included. By comparing the protein-free and the full HDL models, we can clarify the role of lipids and proteins in HDL. The principal objective is to fill the gap of detailed structural and dynamic information of lipids in spheroidal HDL particles. We also address questions related to the role of apoA-I proteins and their interactions with lipids in HDL structures. The currently incomplete understanding of the latter issue is largely due to the size heterogeneity of HDLs (diameters range from 7.2 (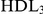) to 12 nm (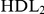)) and the large flexibility of apoA-I. The latter renders the prediction of the positioning of different alpha helices of apoA-I on a spherical surface very difficult. The distribution of lipids inside HDL and their interplay with apoA-I are of profound interest. From a more general perspective, knowledge of the structure of spheroidal HDL is crucial for understanding the conformational changes when HDL makes the transition from discoidal to spheroidal shape, and the trafficking of CHOL and CE through the action of cholesteryl ester transfer protein [43]. Additionally, to design nanoparticles with desired surface and bulk properties, e.g., for controlled transport and release of drugs and contrast agents, it is vital to understand the conformational changes as well as the underlying mechanisms in detail [44]–[51].

## Results

### Structure of Full HDL and the Lipid Droplet

**Figure 3:**
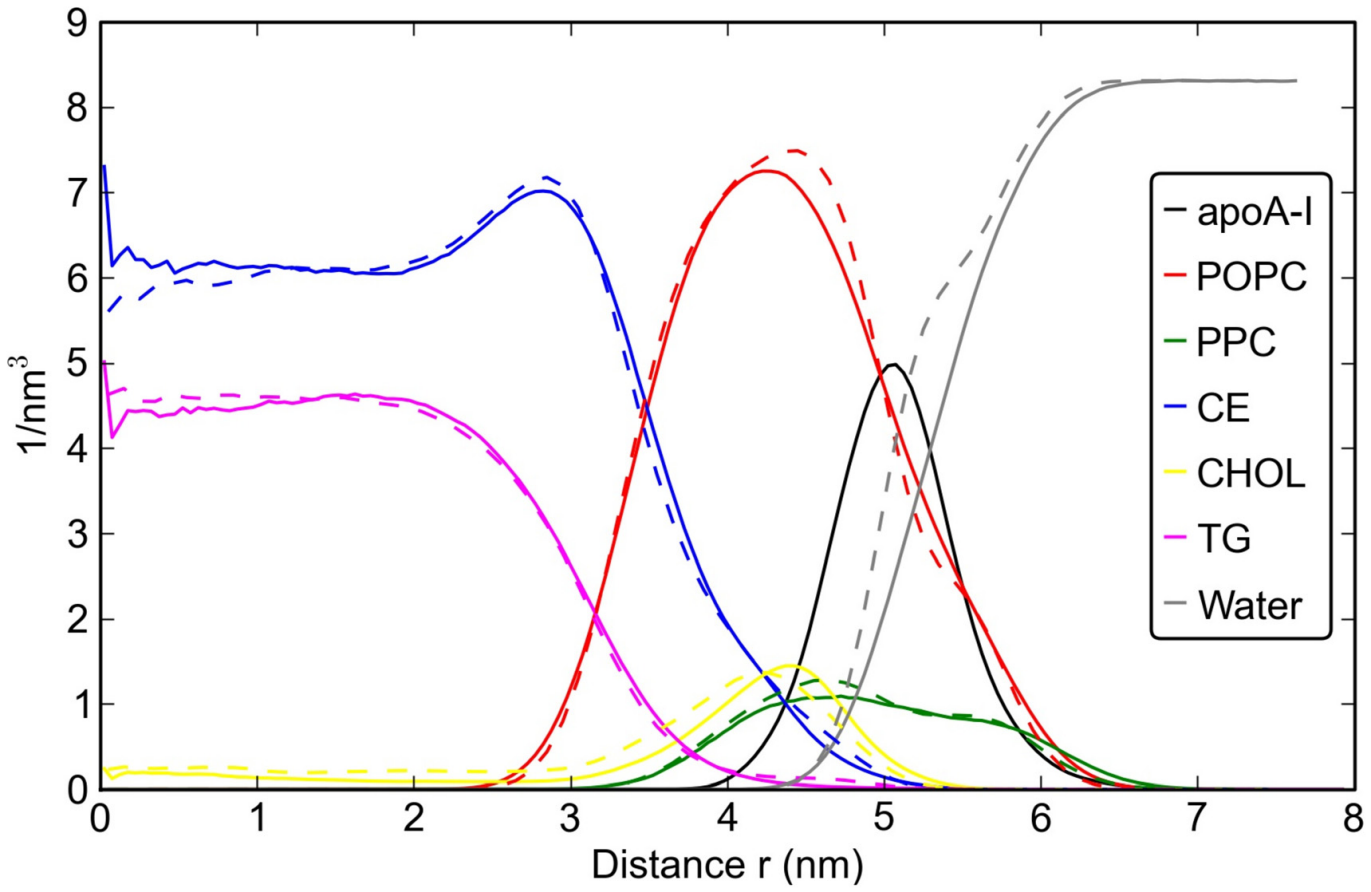
Radial densities showing the composition of the studied particles versus distance from the center of mass (COM) of the particle. The solid lines are for the full HDL particle, the dashed lines for the lipid droplet without apoA-I.

The radial density distributions shown in Figure 3 reveal the internal structure of the simulated lipoparticles. The hydrophobic CE and TG molecules are located in the core of the particle and have minimal overlap with water. The lipids with a polar head group, POPC and PPC, are mostly located at the surface region facing water, whereas most of CHOL is located just below these two lipids. Note that a small but significant concentration of CHOL is also found in the core of the particle.

Considering the size of HDL, the radii of gyration give an average of 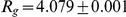 nm for the droplet and 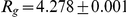 nm for the full HDL. Both particles are effectively spherical, as indicated by the moments of inertia (data not shown).

The apoA-I proteins are embedded onto the surface of the HDL particle, their density peaking just slightly below the headgroup region of POPC and PPC. The presence of the protein slightly disturbs the distribution of these lipids as revealed by the comparison of the lipid droplet with the full HDL particle. The distribution of hydrophobic lipids remains undisturbed. Most significant is the shifting of the distribution of CHOL, and partly PPC, towards water phase when the protein is present, while the distribution of POPC is shifted slightly towards membrane center, making room for CHOL and PPC. In the full HDL particle, water is found to distribute less to the particle compared to the droplet.

Our results clearly highlight the displacement of CHOL even further towards the interface in the full HDL particle. The data below shows that CHOL interacts prefentially with some of the protein residues, strongly promoting the partitioning of CHOL to the vicinity of apoA-I. CHOL further prefers to reside next to the water region, facilitating (hydrogen) bonding via the polar OH group. It has been proposed [52] that CHOL molecules can mediate the relief of membrane stress arising from chain-chain mismatches, since their dimerization is not favored in membranes with high surface curvature. This view is supported by the observations of Huang and Mason [53]. Their work suggests that high surface curvature requires CHOL to be at the interfacial region. Interestingly, Lemmich et al. have further found that very small amounts of CHOL (less than about 3 mol-%) may soften the interface and hence promote its fluidity [54]. In HDL, the average concentration of CHOL is about 10 mol-%, but at the interface it is about 5–10 mol-% depending on distance from the water phase (see Figure 3).

The minor but significant concentration of CHOL in the core of the particle calls for discussion. The usual assumption especially in studies of lipid membranes is that CHOL resides at the water-lipid interface due to its polar OH group. This is expected often to be the case, though there are also reported exceptions such as CHOL residing for short times in the middle of a polyunsaturated lipid bilayer [55], [56], and the suggestion of CHOL in the interior of LDL [57].

**Figure 4:**
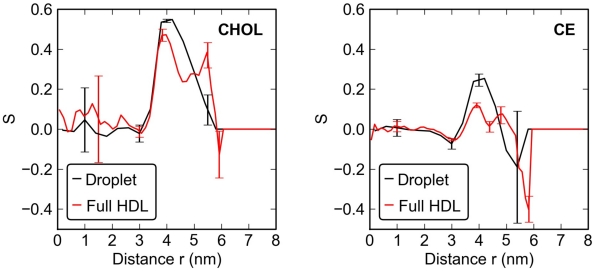
Order parameter 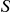 for the ring structures of CHOL (left) and CE (right). The black curves describe the lipid droplet and the red curves the full HDL.

To start with, one gets an impression that the density plot adheres to the two-layer model [6], [7], [22] wherein one assumes almost full separation of hydrophilic and hydrophobic molecules into two separate regions. While the distribution of TG fits into this picture, the distribution of CE and CHOL does not. A rather significant amount of CHOL is also in the core of the particle as was discussed above. Detailed consideration further reveals that there is a significant overlap of CE with CHOL, POPC, and PPC: The radial density distributions shown in Figure 3 do not provide a sufficiently unique description of only two different structural regions inside HDL. Furthermore, by looking at the order parameters of CHOL and CE presented in Figure 4 it becomes evident that there are not only two regions but also the intermediate one between the hydrophobic and hydrophilic ones. The innermost *core* of the particle (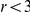 nm) is clear, as there the ring structures of both CHOL and CE are oriented in a completely random fashion. The situation changes as one approaches the lipid-water interface through the *intermediate* region (3 nm 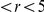 nm), which is characterized by significant ordering of the ring structures, in a manner where the principal axis of CE's and CHOL's ring moiety lies along the radial direction of HDL. This intermediate region overlaps with the distribution of the acyl chains of POPC and PPC, revealing that the sterol rings are also aligned with the acyl chains. Finally, at the HDL-water interface, one finds the region composed of hydrophilic headgroups of POPC and PPC that constitute the *surface* part of the lipid droplet interacting mostly with water.

The data clearly shows that instead of the two-layer model, the distribution of lipids in HDL is more complex. The structural nano-scale organization of CHOL and CE plays an important role in constituting the intermediate layer. However, there is no apparent reason to conclude that the lipid droplet in HDL would be described by a “three-layer” model either, since the intermediate region is narrow and represents a crossover from the hydrophobic to the hydrophilic environment rather than a clearly defined layer of its own. Our results for lipid dynamics are in favor of this view and will be discussed below in the context of diffusion. Meanwhile, while quantitative results have been missing, a three-layer model has earlier been proposed for LDL particles [3]. There the situation is different, though, since the diameter of LDL is roughly three times larger compared to HDL and the intermediate region can possibly be broader and more characteristic compared to HDL.

There are significant differences when the order parameters of CHOL and CE are compared (Figure 4). First, the height of the main peak is considerably lower for CE than for CHOL, indicating that the ring of CE has a lower tendency to orient itself along the acyl chains than CHOL. Second, unlike for CHOL, on the surface of the particle (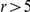 nm) the order parameter of CE obtains negative values. These indicate the ring of CE to lie along the surface, perpendicular to the radial direction. This obviously stems from entropic reasons, since while CE strives in part to organize its structure like CHOL, also directing its weakly polar ester bond region to the surface like CHOL does for the OH group, CE also has a long oleate chain. Previous atomistic simulations of CE in bulk conditions as well as in a combined system with POPCs have shown that the oleate chain of cholesteryl oleate has essentially three different conformations with respect to the ring of CE [33], [36]: one where the angle of the oleate chain (describing it as a semi-stiff rod) with respect to the principal axis of the ring is about 35 degrees, and two other modes with an angle of 90 or 150 degrees. Compared to CHOL with only one mode, CE inevitably aims to minimize free energy by promoting entropic degrees of freedom.

Another interesting observation is that apoA-I suppresses the main peak of both CHOL and CE molecules in Figure 4. The effect is an indication that the protein disturbs the ordering within the intermediate region (between the core and the surface), also facilitating the displacement of CHOL towards the water phase. This conclusion is supported by the broadening of the angle distributions of POPC in the presence of the protein (see Supporting Information (SI)).

**Figure 5:**
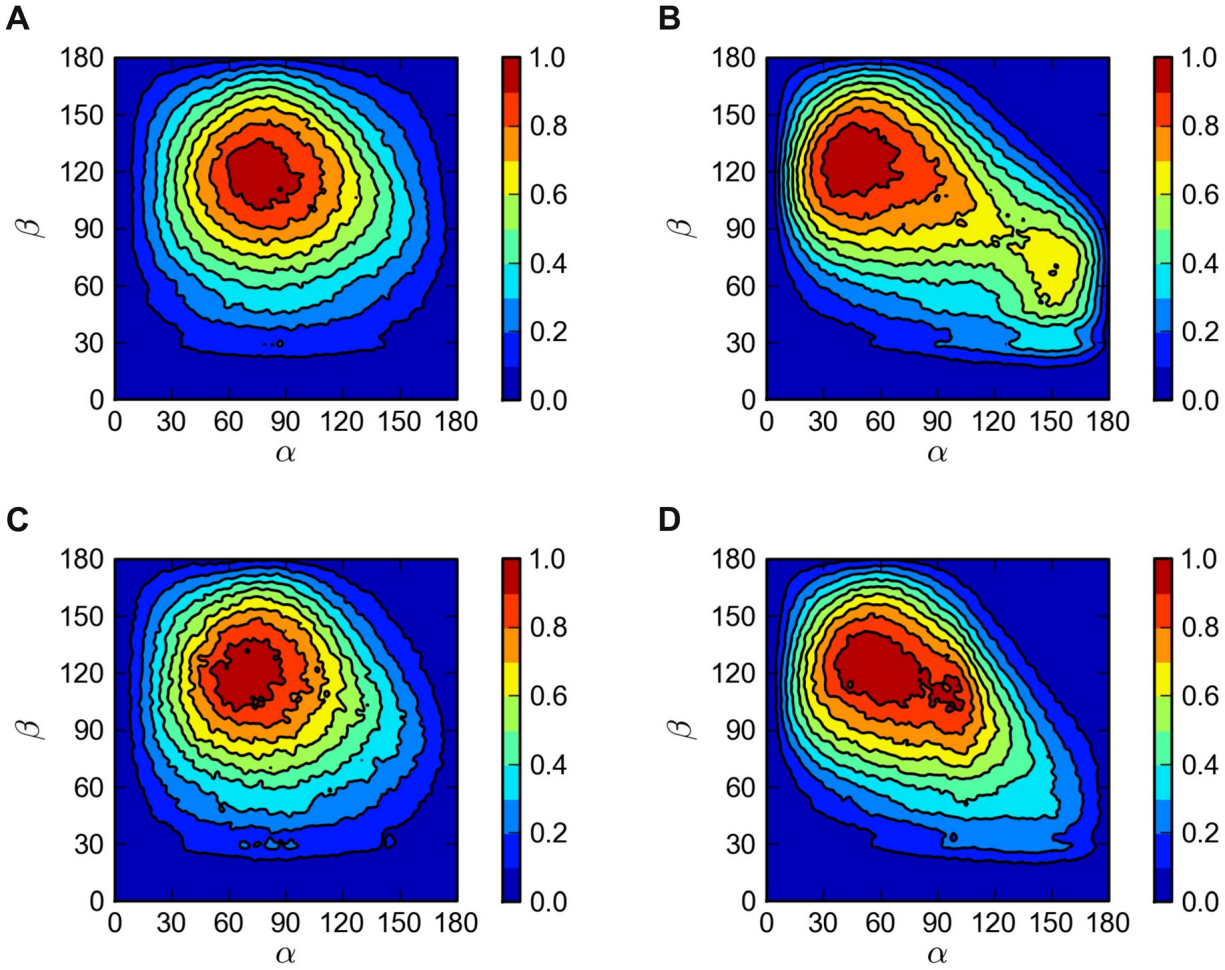
Distributions of CE conformations. The horizontal axis is the angle 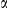 between the CE ring and the effective normal of the lipid droplet. The vertical axis is the angle 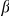 between the ring structure and the oleate chain. The left panels (A, C) describe the core of the droplet (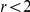 nm) and the right panels (B, D) the surface (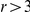 nm). The pictures at the top (A, B) show the lipid droplet without apoA-I and those at the bottom (C, D) the full HDL.

An analysis of the internal conformations of CE molecules in Figure 5 provides a more detailed view of the situation. In the core of the particle, the most probable conformation of CE is the coil-like conformation (maximizing entropy), where the angle between the CE ring and the oleoyl chain is about 120 degrees. This is largely consistent with recent atom-scale simulations of CE in bulk conditions [33]. The behavior changes on the surface of the particle. The two peaks of the distribution on the surface correspond to two distinctly different conformations: one where the ester group of CE (corresponding to the OH-group of CHOL) points towards water and the oleate chain is extended towards the solvent, and another where the ester region is pointing towards the core of the particle, while the ring and the oleoyl chain form a small angle with each other.

Also for TG, we find a change of conformation when it is shifted from the core of the particle onto the surface. In the core, the three chains of TG place themselves to a similar conformation as in a bulk melt of TG [34]. When brought to the surface, the ester bond regions seek contact with water, which brings the three chains of TG closer to each other into a more tightly packed conformation (see Figure S2). Additional data for molecular conformations are presented in Figure S1, Figure S3, Figure S4, and Figure S5.

### Dynamics of Lipids Characterized by Diffusion

The large-scale dynamics within HDL and the lipid droplet are considered in terms of diffusion, characterized by the diffusion coefficient 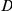. The diffusion coefficients were determined by considering lipid displacement distribution functions over a fixed period of time (see SI). We found that the jump length distributions for lipids in the core of the particle (TG and CE) fitted well with the three-dimensional diffusion model, yielding 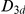. Meanwhile, the lipids on the surface (POPC, PPC, CHOL) fitted much better with the two-dimensional description for diffusion, yielding 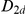. For details, see SI.

Table 2 shows the long-time diffusion coefficients of the lipid components within the lipid droplet and the full HDL particle.

**Table 2:** Diffusion coefficients in units of 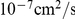.

Component	Dimensionality	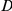 (droplet)	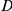 (HDL)
TG	3d	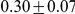	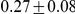
CE	3d	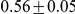	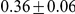
CHOL	2d	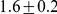	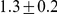
POPC	2d	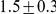	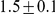
PPC	2d	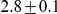	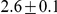

The dimensionality of 2d refers to diffusion along the lipid-water interface, while 3d refers to diffusion in the core of the particle.

**Figure 6:**
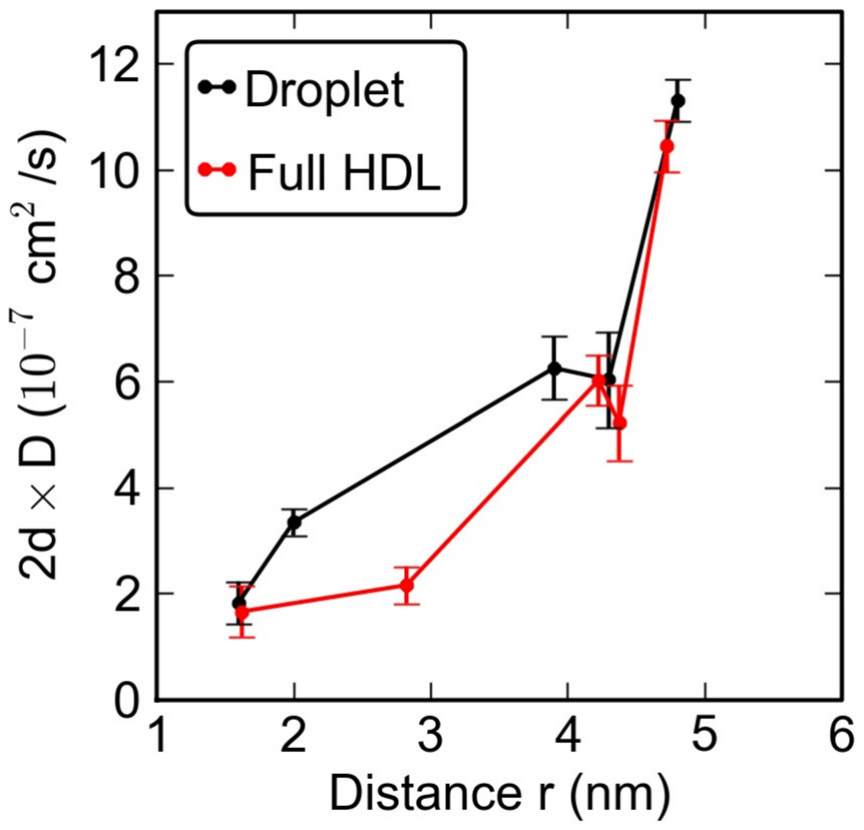
Diffusion coefficients of the lipid components. Each point in the plot describes the diffusion coefficient for one of the lipid types. The distance 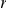 is the average distance of the given lipid from the COM of the particle. To facilitate comparison between core (three-dimensional diffusion) and surface lipids (two-dimensional diffusion), the coefficients have been scaled with 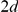, where 
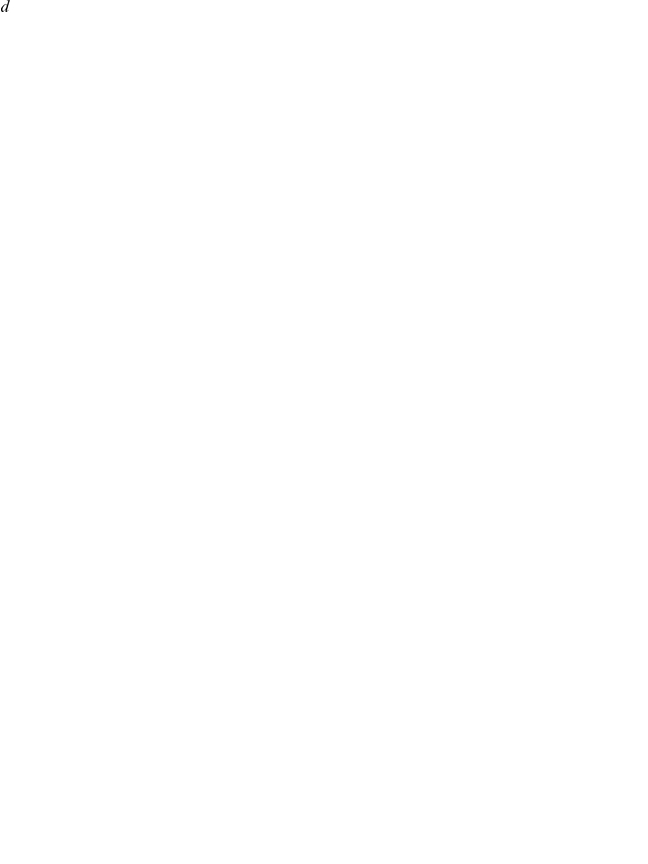
 is the dimension of the fit (either two or three).

Figure 6 depicts how the diffusion rate varies significantly inside the lipid droplet and/or full HDL. The diffusion is the slowest in the middle of the particle, it speeds up as the molecules get closer to the interface, and the fastest diffusion is found at the interface. The influence of apoA-I on diffusion of lipids is modest. It turns out that the lipid diffusion coefficients in the protein-free lipid droplet and the full HDL particle are almost similar. The apoA-I proteins may slow down the diffusion of lipids slightly especially close to the interfacial regions. The effect is, however, weak (see Table 2).

The diffusion coefficients of POPC, PPC and CHOL in the surface region of the particles are about 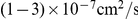 and in good agreement with experimental estimates of 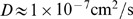 for two-dimensional lipid bilayers in fluid phase [58]. On the other hand, the diffusion coefficients for CE and TG are smaller by a factor of 10, about 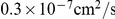. To our knowledge, diffusion coefficients of lipids in HDL have not been experimentally determined. However, for LDL and LDL-like lipid droplets, Vauhkonen et al. used pyrene-linked PC lipids as probes to find that 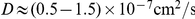 at the surface of lipoparticles [59], in good agreement with our findings. Massey and Pownall have further used single-chain cationic amphiphiles for considering lipid mobility at the surface regions of LDL and HDL, and while quantitative estimates for 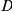 are missing, they concluded that the diffusion at the surface is about 2–3 times slower compared to cholesterol-free POPC vesicles [60]. Recent MARTINI-model simulations for single-component PC bilayers have yielded 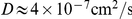 [61], which is about a factor of 2 larger than diffusion at the surface of HDL. While the comparison of our simulation data and experiments is suggestive rather than conclusive, the qualitative agreement is striking.

Our main result regarding diffusion is that diffusion at the surface region of HDL is largely similar to diffusion of lipids in cholesterol-containing lipid bilayers in the fluid-like phase, the cholesterol concentration being roughly 10 mol%. Figure 6 also shows convincingly that the effect of apoA-I on diffusion of lipids is not significant.

Additionally, Figure 6 provides compelling evidence that the dynamics of lipids in terms of their diffusion properties is not consistent with the two-layer model. Instead of two clearly different dynamic regions, we find the diffusion coefficients to increase monotonously: diffusion rates are clearly different in the core (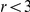 nm), in the ordered intermediate region (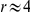 nm), and at the surface (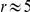 nm).

Given the different proposed models for lipid distribution in HDL, the striking difference of the present findings compared to earlier studies is the role of dynamics: not only the structural and ordering properties of molecules in HDL differ across HDL, but also the dynamics in terms of molecular transport coefficients varies significantly in the different compartments. The biological relevance of this feature lies in the time scales of molecular trafficking inside HDL: while molecular transport between the surface and the intermediate region is relatively fast, the transport between the surface and the core of HDL is slower by a factor of 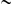
10.

### Role of ApoA-I in HDL

The above results show that the apoA-I proteins do not induce large changes to the lipids' properties inside the droplet. Yet, while the protein collapses onto the surface of the droplet, it does disturb the packing, ordering and, although only slightly, also the dynamics of the lipids at the surface region. What remains to be explored is the nature of the lipid-protein interactions. In this work our primary interest is the lipid component of HDL, thus we have used the standard CG MARTINI model which does not enforce the full secondary structures in apoA-I. This optimizes computational efficiency and allows us to focus on generic issues such as the partitioning of lipids around apoA-I, and the influence of apoA-I on the lipid droplet. Meanwhile, we cannot address questions related to detailed atomistic phenomena at the lipid-apoA-I interface.

Data for the surface accessible surface areas (SASAs) of apoA-I hydrophilic and hydrophobic residues (data not shown) provide evidence for the low contribution of protein hydrophobic residues (11%) to the total SASA of the protein, the main contribution coming from protein hydrophilic residues (89%). The average value of SASA of protein hydrophobic residues (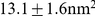) is in good agreement with that reported by Shih et al. [32] in a recent study on the assembly of lipids and proteins into lipoprotein particles.

**Figure 7:**
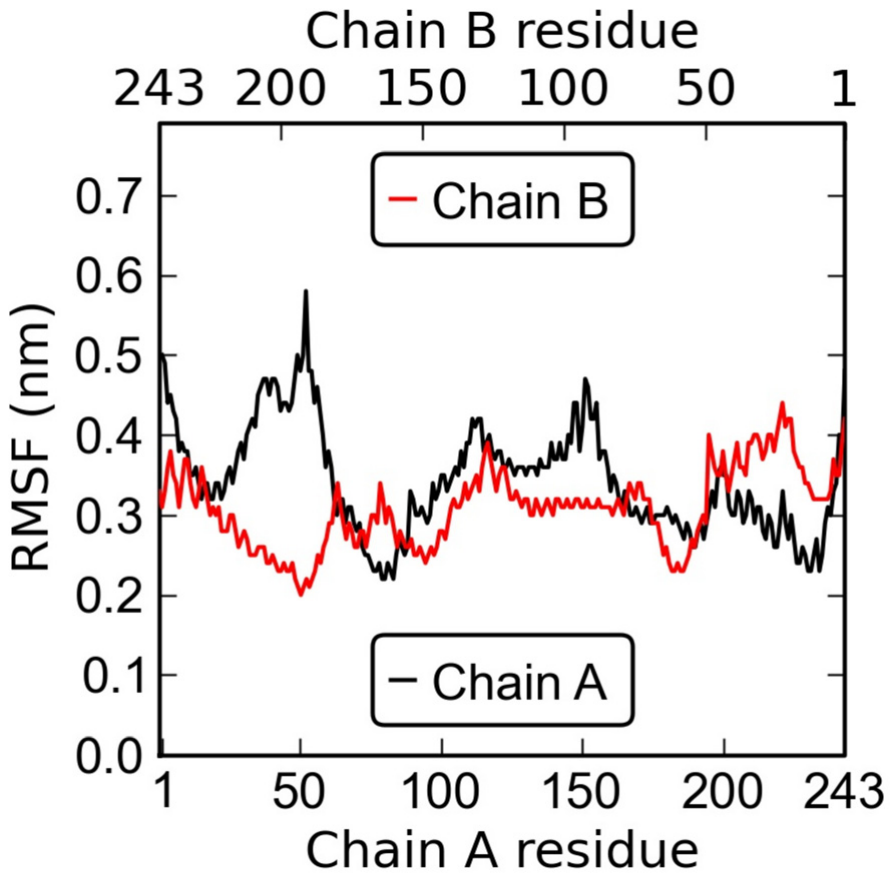
Root mean square fluctuation (RMSF) profiles for apoA-I alpha carbons (black, chain A; red, chain B) measured over the last 4 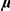
s of the simulation of the full HDL particle. Experiments suggest that the alpha-helical region is likely given by the residues 44–241, and that the alpha-helical content overall is about 75–80% [6], [13], [18], [26].

The RMSFs of protein 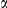 carbons are shown in Figure 7 and reveal the mobility of different protein domains. The 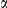-helical structure of the protein exhibits very little mobility for both chains. This rigidity of the protein is also in agreement with the observed slight disturbances produced on the lipid packing.

**Figure 8:**
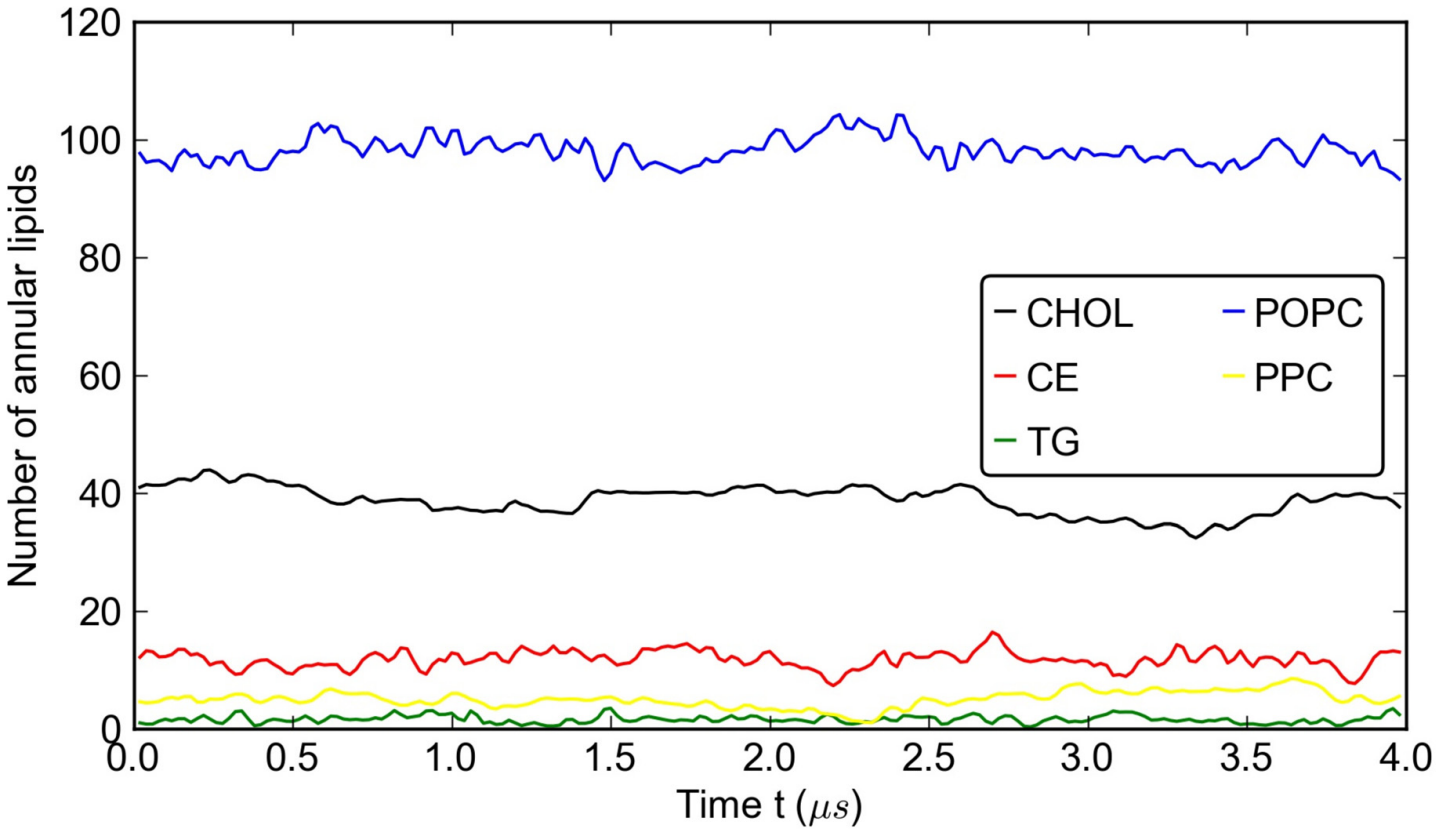
Number of annular lipid molecules over the last 4 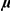
s of the simulation of the full HDL particle: POPC (blue), CHOL (red), CE (orange), PPC (purple) and TG (green).

The number of annular lipids, as defined in the Method section, is given in Figure 8 for each lipid component. It is interesting to note that about 80% of CHOL molecules are annular (on average 40 out of 49) while only 10% of CE molecules (about 15 out of 122) are in close contact with the protein. An average of about 98 POPC molecules out of a total of 260 are annular. Overall, the results indicate that there is a preferential interaction between CHOL molecules and protein residues. This result is striking if one considers that the number of POPC molecules is larger than that of CHOL molecules.

It is known that the number of apolipoproteins in HDL depends on particle size. We characterized its role for lipid distribution through additional simulations with three apoA-Is. First, we performed a 20 microsecond simulation of the same lipid droplet with 3 apoA-I molecules placed 2 nm apart from each other. The protein molecules were found to insert themselves in the lipid droplet in the same way as was observed above, with hydrophobic moieties pointing towards the droplet. The only interesting difference was that in the structure with three apoA-Is, the C-terminus and the helix 9 of one protein molecule were not inserted in the lipid droplet. This situation is likely due to the crowded arrangement of apolipoproteins, or due to the limited time scale of the simulation. The addition of a third apoA-I molecule does also affect the interaction of cholesterol with apoA-I: Almost 100% of the cholesterol molecules (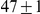 out of 49) are in contact with the three proteins. That is, the addition of the third apoA-I molecule enhances the average number of annular cholesterol molecules from about 80% (observed with 2 apoA-I molecules) to about 96% of the total unesterified cholesterol in the particle.

**Figure 9:**
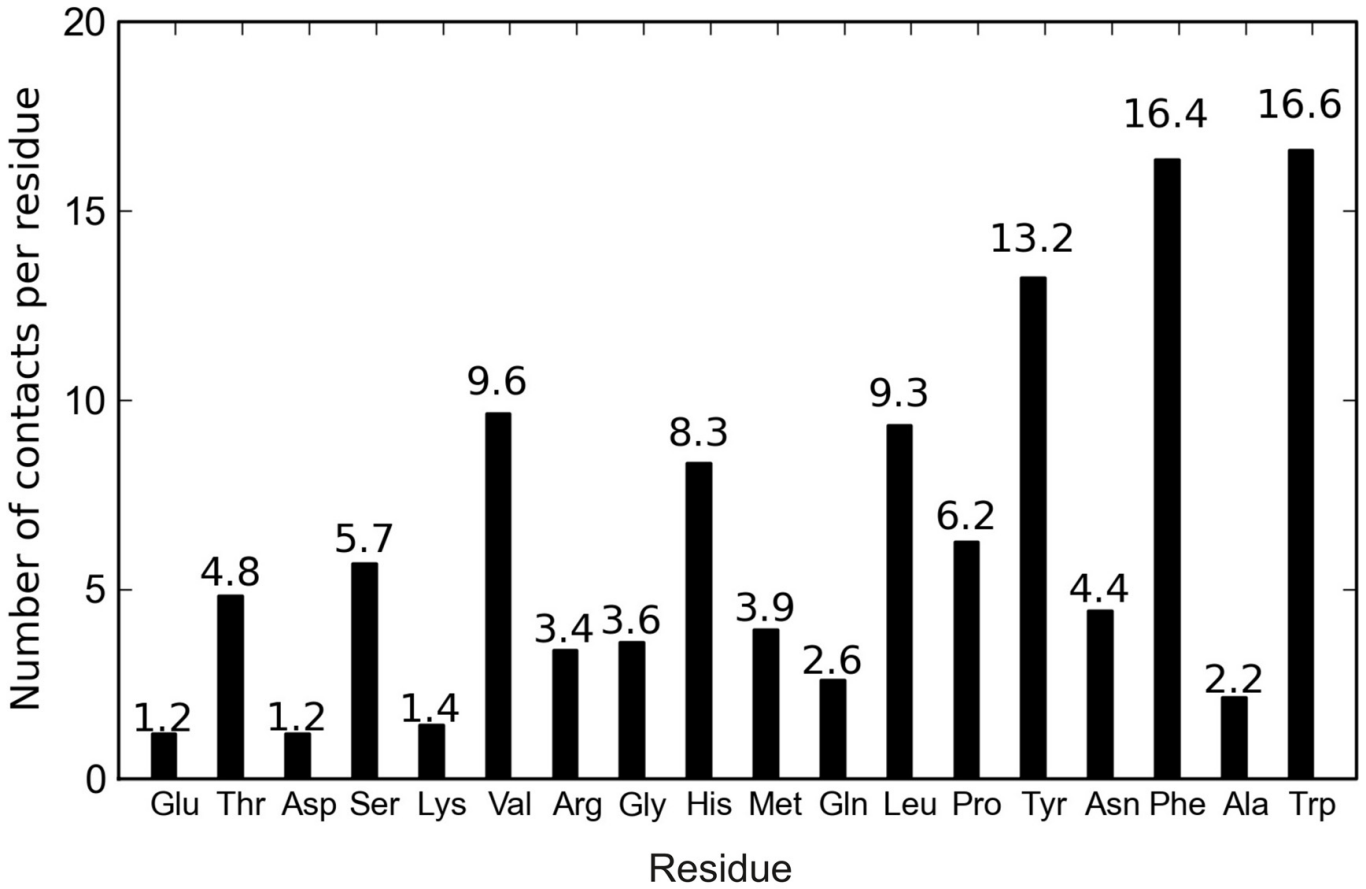
Number of contacts of CHOL molecules with apoA-I residues normalized per residue. See text for details. Isoleucine and cysteine are absent in the human apoA-I sequence considered here [13].

The lipid-protein interactions of different moieties of each lipid component showed that POPC, PPC and TG molecules interact with apoA-I residues preferentially through the acyl chains (POPC and PPC glycerol backbone has also a high number of contacts with apoA-I), while CHOL and CE molecules interact with the protein mainly through the sterol ring (see Table S1). To better understand the nature of the interaction between CHOL and apoA-I we also measured the number of lipid-protein contacts per residue (hydrophobic and hydrophilic), shown in Figure 9. It is clear that there is a preferential interaction of CHOL molecules with apoA-I hydrophobic residues, in particular tryptophane (Trp) and phenylalanine (Phe) having aromatic side chains, but also valine (Val) and leucine (Leu). Highly preferred interaction with Trp and Phe is understandable through findings of aromatic ring pairing in e.g. known protein structures [62]. We also observe a relevant number of contacts with apoA-I hydrophilic residues with aromatic side chains such as tyrosine (Tyr) and histidine (His). This is not surprising, as Tyr has a hydrophobicity comparable to Phe as has been shown experimentally by Wimley and White [63] through the determination of a hydrophobicity scale for proteins at membrane interfaces. There are less contacts of CHOL molecules with the other apoA-I hydrophilic residues, namely serine (Ser), threonine (Thr) and asparagine (Asp) being the most attractive ones. These results are in good agreement with the observed large number of contacts of the sterol ring of CHOL molecules with protein residues.

**Figure 10:**
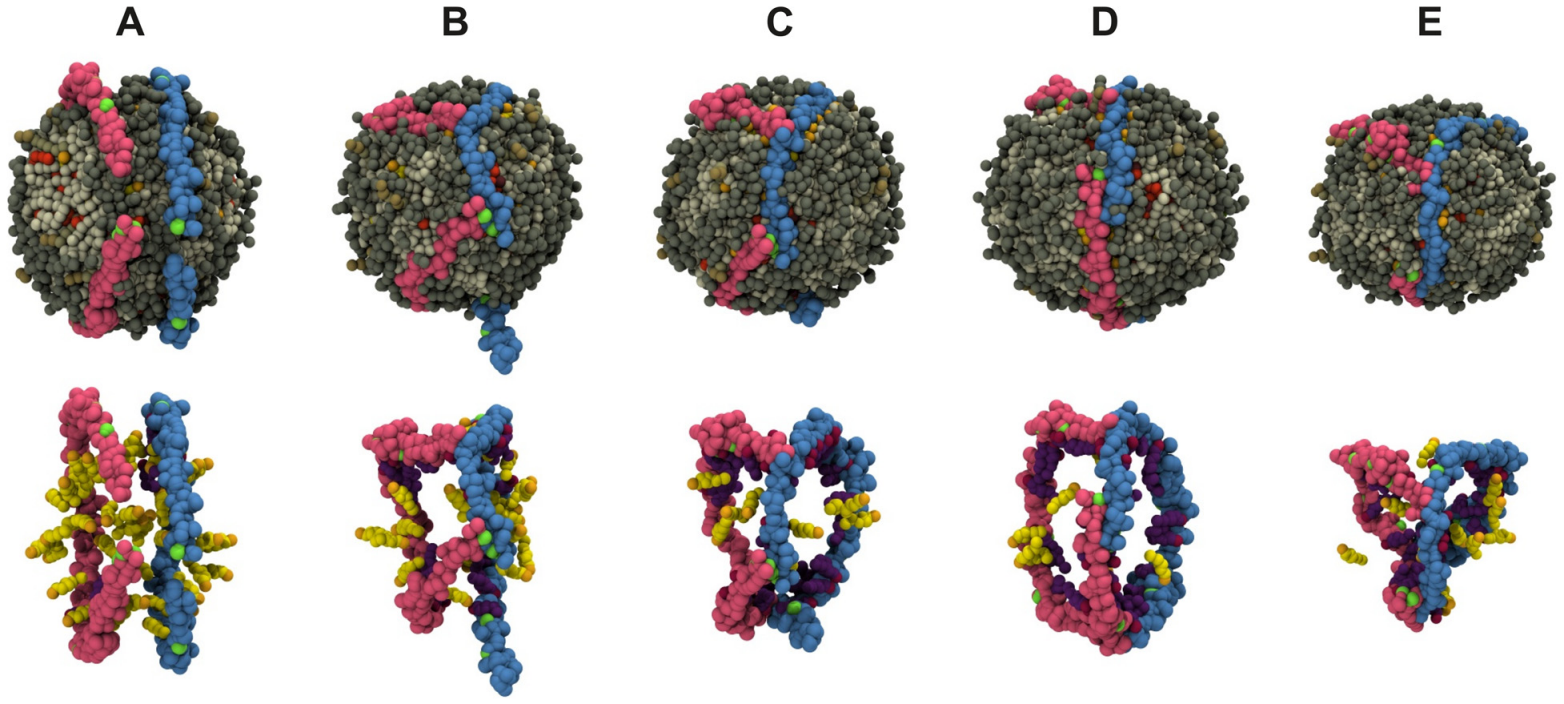
Illustrative snapshots of HDL structure. (Top) Different snapshots of the full HDL simulation: (A) 0 
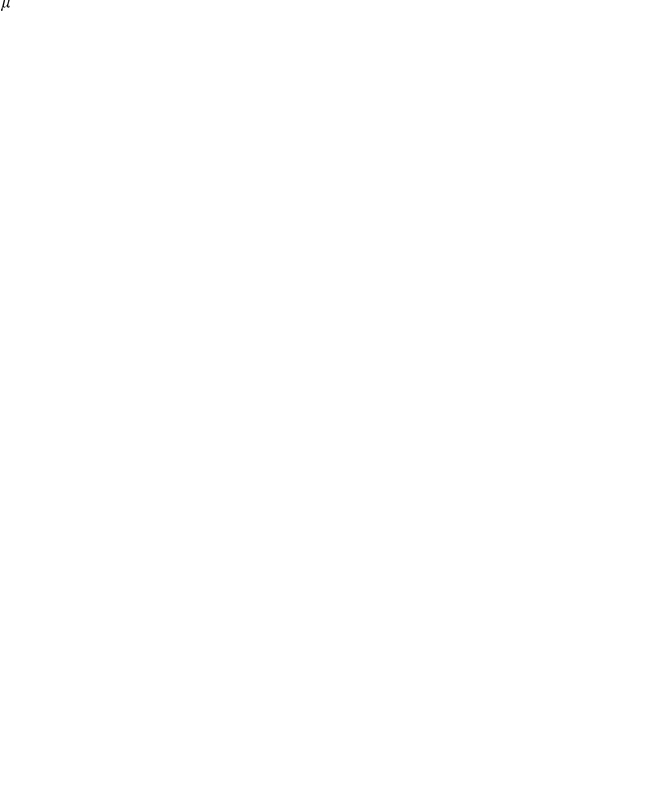
s, (B) 0.4 
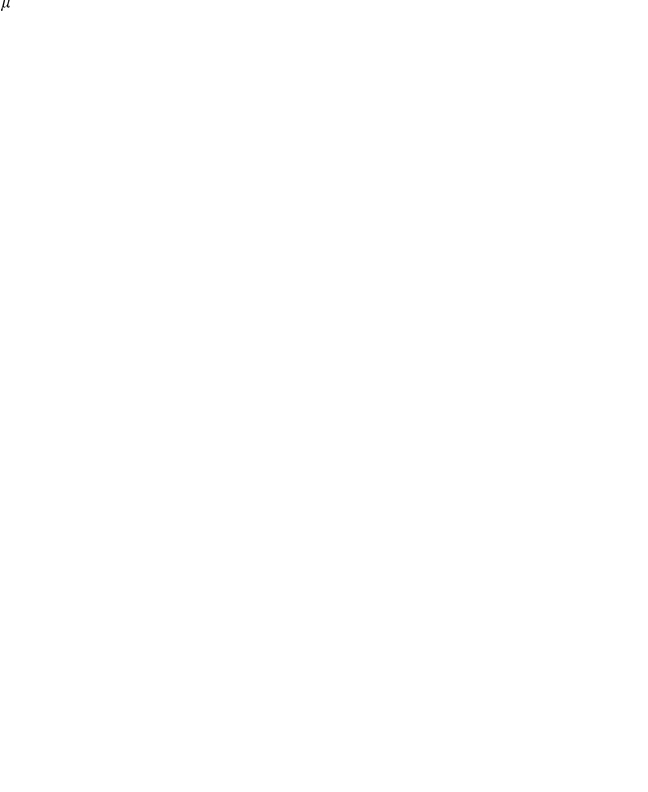
s, (C) 1.4 
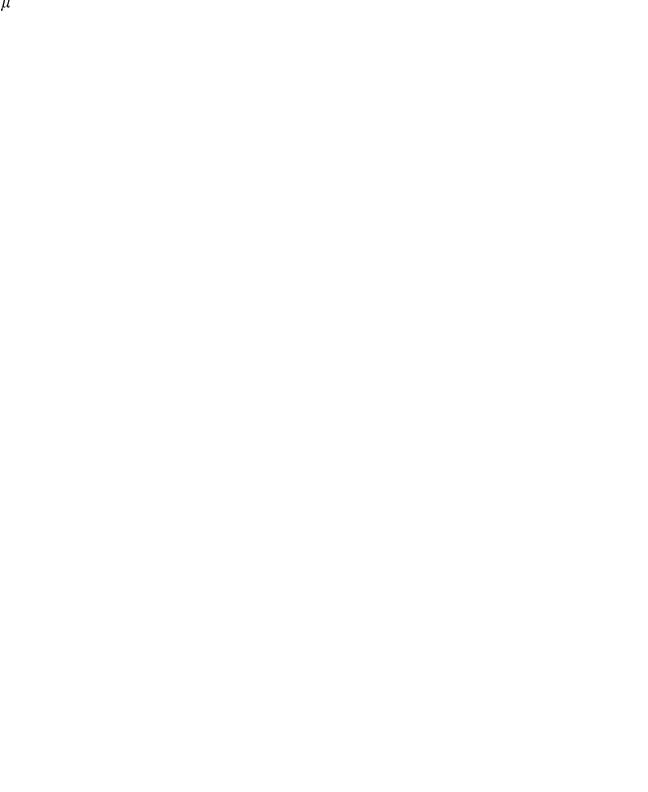
s, (D) 12.4 
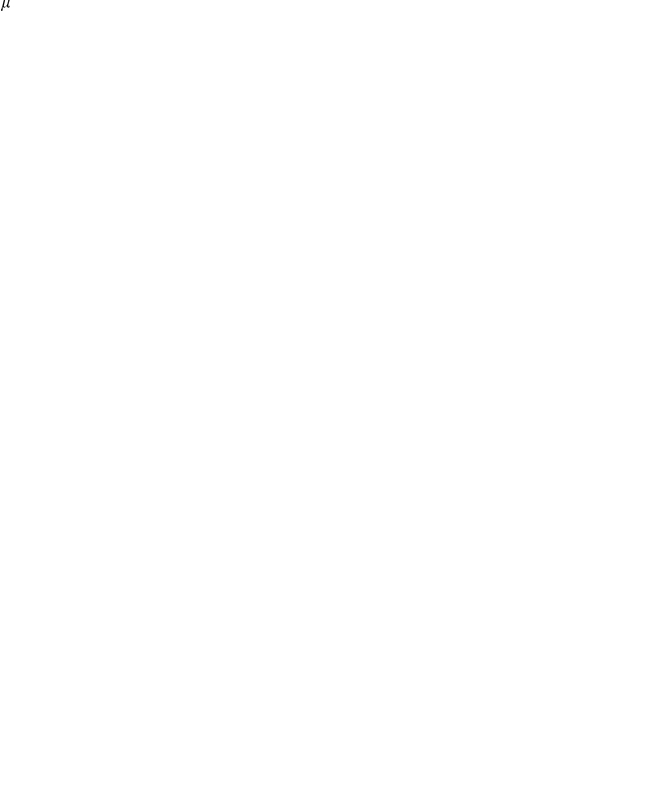
s, and (E) 19.04 
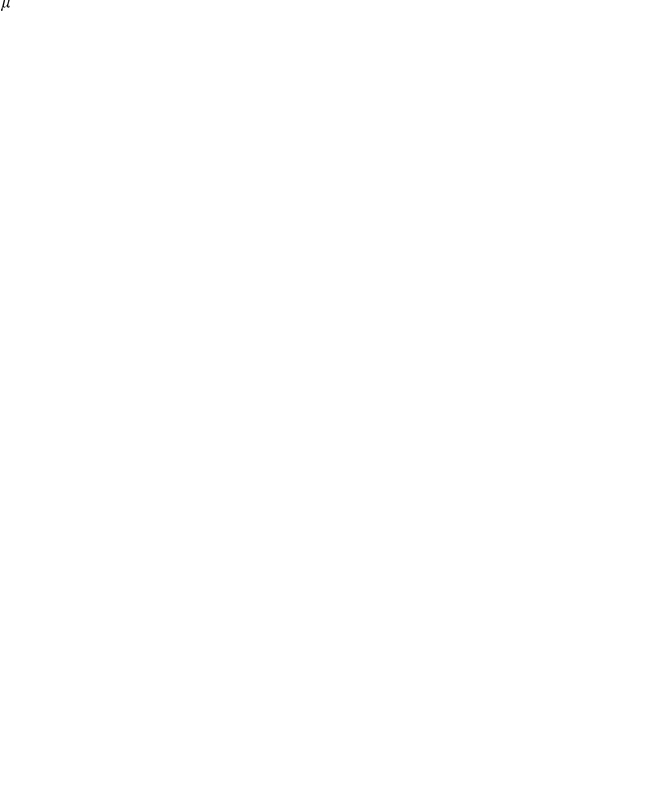
s. (Bottom) Snapshots displayed at the top of the figure showing here only the apoA-I molecules, and annular and bulk CHOL molecules. The two apoA-I chains are in light red (chain A) and light blue (chain B) with proline residues in green. Annular CHOL molecules are shown in purple with a dark red hydroxyl group. Bulk CHOL molecules are depicted in yellow with an orange hydroxyl group.

The sterol ring of CHOL molecules can intercalate or interact with the aromatic side chains of protein residues as observed for CE in a recent study by Catte et al. [36]. This interaction between CHOL molecules and apoA-I was also observed experimentally by Dergunov et al. [64]. The authors estimated the degree of exclusion of CHOL molecules from the boundary lipid region in reconstituted discoidal HDL particles containing different apolipoproteins and observed an increase in the order A-I<E<A-II. The partial exclusion of CHOL molecules operated by apoA-I and the corresponding CHOL distribution among surface and bulk lipids are in good agreement with our findings as depicted through a series of snapshots in Figure 10 (see also SI).

The binding between CHOL molecules and apoA-I residues is quite weak, which permits exchange among apoA-I -bound and free CHOL molecules on the time scale of the simulation. We characterized this trafficking process by computing the distributions of lifetimes between CHOL-protein and CE-protein contacts. The average lifetime was found to be 146 ns for CHOL and 15 ns for CE. While the errors are of the same order as the lifetime due to a limited number of samples, and the fact that the distribution for CHOL is broad as there are cases where the CHOL-protein contact is maintained throughout the simulation, the results highlight the stability of CHOL-protein binding with respect to that of CE. The relatively large lifetime of the CHOL-protein binding also highlights that once CHOL has migrated to the vicinity of apoA-I, it remains there for a long period of time. For comparison, the average non-contact lifetime for CHOL-protein pairs, describing the characteristic time for CHOL to not be in contact with any parts of apoA-I was found to be about 175 ns. That is, CHOL molecules reside close to the water-HDL interface and on average spend half of their time in contact with apoA-I.

The above results are in good agreement with NMR experiments performed on human HDL, which indicate that CHOL molecules are present in two distinct environments [65]. More specifically, Lund-Katz et al. found that the cholesterol molecules dissolved in the core of HDL are relatively disordered and mobile, while the cholesterol molecules located among phospholipid molecules in the surface of the particle undergo relatively restricted, anisotropic motions. This view is in line with our simulation results discussed earlier in this article. Lund-Katz et al. thus proposed that cholesterol molecules are in two different microenvironments, undergoing fast exchange between these two regions, equilibrating between the surface and the core of HDL in the time scale of milliseconds or more. While the time scales proposed by Lund-Katz et al. are beyond those that are accessible via simulations, we have found that there is local exchange taking place at times up to microseconds. However, the time scales we have found via simulations should be regarded as the lower limit, since the diffusion coefficients we have found for the core of HDL imply that the exchange of cholesterols between the core and surface regions has to be larger than 1 
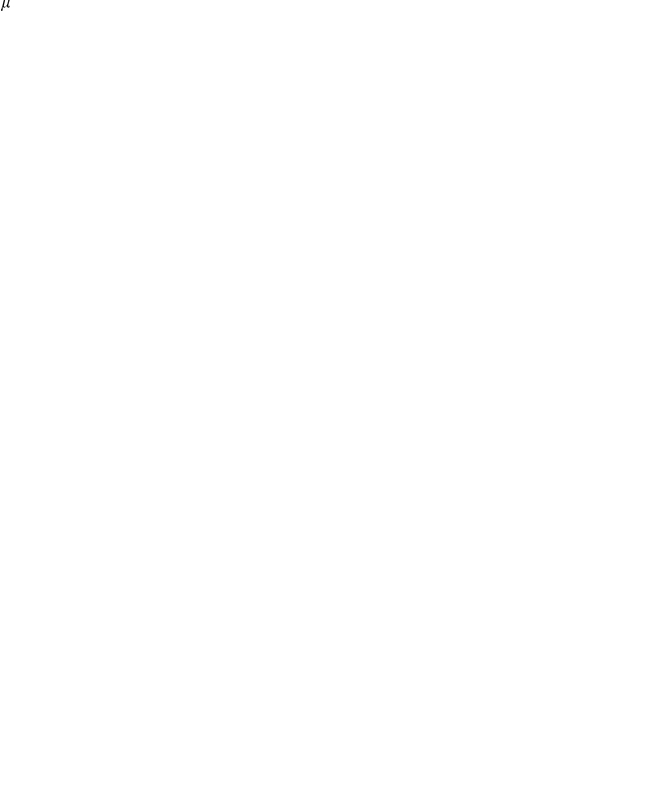
s.

## Discussion

In this study, we elucidated the structure and dynamics of spheroidal high density lipoparticles with a realistic lipid composition corresponding to human serum HDL. We found that the traditional two-region model for HDL is not accurate enough. Instead, we found the distribution of the different lipid types in HDL to be more complex.

The innermost core of HDL is mainly occupied by TG and CE, which as hydrophobic lipids constitute a randomly oriented melt. However, in contrast to the common view, the inner core was also found to contain a rather significant fraction of free cholesterol partitioned into the disordered melt. The outermost surface region constitutes the interface with water, which is mostly occupied by the polar headgroups of POPC and PPC. Between these two is the intermediate region occupied by CHOL, partly also CE, and the acyl chains of POPC and PPC. However, there is no apparent reason to consider the intermediate region as a “third layer”, since it is narrow, unclear to define spatially, and represents a crossover from the hydrophobic to the hydrophilic environment rather than a true layer of its own. Yet it has properties that are distinct from those in the core and at the surface.

This is most obvious in two aspects: ordering of steroid moieties and molecular diffusion. Unlike in the core, in the intermediate region the bulky rings of CHOL and CE are strongly ordered along with the acyl chains. This ordering extends also to the surface region of HDL, highlighting the difficulty to define the intermediate region as a true layer of its own. This view is also supported by the diffusion data, which illustrates that the diffusion of molecules takes place at a clearly different pace in the different regions. In the core and in the intermediate region of the particle, diffusion was found to be three-dimensional, while the diffusion of lipids at the HDL-water interface turned out to be two-dimensional in nature. Quantitatively, diffusion in the core of the particle was observed to be slow, as in a polymer melt, and to speed up monotonously as one crosses the intermediate region and ends up in the interfacial region.

The perspective arising from our results is novel, providing the first molecular scale view to the nano-scale organization of lipids in HDL. The present results indicate that the spatial distribution of lipids within HDL provides only a narrow perspective to the complexity of lipid organization. To understand this issue, one has to pay considerable attention not only to density distributions but also conformational and orientational degrees of freedom of the lipids, and their dynamics within HDL.

Events where CE and TG penetrate to the surface were found to be rare. In the few observed cases when it occurred, their conformation was significantly changed. In the core of the particle, both CE and TG were observed to be capable of obtaining more coil-like conformations. This indicates that the formation of the HDL core is not only driven by the hydrophobic effect, but that conformational entropy has a significant effect.

When comparing the simulation of the full HDL (with apoA-I) to the lipid droplet (without apoA-I), we found that the overall structure of the lipid droplet was not significantly changed by the presence of the protein. Rather, we found a disturbance in the behavior of the surface lipids. In particular, the order of CHOL and CE molecules decreased and the conformations of the acyl chains of PC lipids got broader. Diffusion of the surface lipids was slightly perturbed by the protein, but the effect was minor.

The low contribution of the SASA of apoA-I hydrophobic residues to the total SASA of the protein and the large number of contacts of hydrophobic moieties of each lipid component with apoA-I evidence that the hydrophobic forces drive the insertion of the protein and contribute to the stability of the full HDL. Interestingly, we found that a large number of CHOL molecules interact with apoA-I, mainly through their sterol ring and especially with hydrophobic residues having an aromatic side chain. We also observed fast exchange among protein-free and protein-bound CHOL molecules. This result is in good agreement with experimental findings for human HDL particles [65].

It is tempting to discuss the physiological relevance of the above-discussed molecular level findings, especially the preferable interaction of the sterol ring moiety with the aromatic amino acids, and the observation that CHOL molecules spend about half of their time in contact with apoA-I, trafficking relatively rapidly back and forth in the vicinity of apoA-I. Such interaction is prone to have impact on the availability of sterols and lipids for related transfer proteins and enzymes, such as the cholesteryl ester transfer protein (CETP) and cholesteryl esterases. This interaction may be even more important in the process of cholesterol efflux, which is the critical part of reverse cholesterol transport, where the accumulated cholesterol is removed from macrophages.

For future purposes for characterizing the properties of HDL, as well as HDL under enzymatic reactions, our results bring about a useful view to consider. We have found that the interfacial region of HDL close to the water phase is rather well defined in terms of its molecular composition: it can be described as a model layer composed of PCs, lyso-PCs, CHOLs, and the apolipoproteins A-I. The diffusion results discussed in this study indicate that the lateral diffusion along the interfacial layer is largely consistent with diffusion taking place in model membranes, whose molecular composition is of the same type. These features suggest that both the physical and chemical properties of HDL could be explored with reasonable accuracy through studies of (planar) model membranes, which are considerably easier to characterize compared to nano-sized HDLs. Clearly, the insight gained in this manner would be limited, since a number of inherent features would be missing, such as the curved nature of the HDL-water interface and its effect on apoA-I. Nonetheless, there is reason for optimism, encouraging experiments and simulations to use model membranes for better understanding of lipoprotein properties, including both HDL and LDL.

The view presented in this article for HDL structure and dynamics paves way to extend the scope of computational studies for HDL, and to gain a much deeper understanding of HDL properties in a number of conditions related to health. For instance, there is reason to assume that the molecular composition in HDL depends to some extent on factors such as diet and lifestyle. In altered HDL the lipid composition can be abnormal due to e.g. dyslipidemia [66]. Characterization of molecular composition of HDL of subjects with varying degrees of health would allow coarse-grained simulation studies of HDL in these subject groups, using the present results as a reference. Preliminary studies in this spirit have very recently been reported and discussed by Yetukuri et al. [67], who found that an elevated triglyceride concentration in low-HDL subjects also affected its distribution in HDL, increasing the concentration of triglycerides markedly at the lipid-water interface next to apolipoproteins. Such results based on large-scale coarse-grained models can further be fine-grained to atomistic description to study the atom-scale features that are relevant e.g. in lipid-protein interactions, and the implications on HDL stability due to reactions of enzymes such as phospholipases. Work in this direction has already been initiated by Shih et al., who recently fine-grained coarse-grained models for matured HDL particles comprised of apoA-Is, phopholipids, and cholesteryl esters [38]. Similar work is in progress for the present HDL models.

Altogether, considering the complexity of HDL, our study highlights the importance of lipid-apoA-I interactions, and in particular the specificity of apoA-I for free cholesterol and its esters. The molecular-scale insight of HDL structure and dynamics found and confirmed in our study largely stems from the ordering and dynamical phenomena taking place close to the HDL-water interface, being in part driven by the interactions between cholesterol and apoA-I. Evidently, they have a prominent role to play in a number of transport processes dealing with cholesterol.

## Methods

### Simulation Models

Construction of the models was implemented in two stages. First, we constructed lipid droplets (without the apoA-I proteins) using coarse-grained descriptions of lipids and water. Second, the studies of pure lipid droplets were complemented by models where the droplet was surrounded by two apoA-I proteins. Below, we describe the main stages of the model construction.

The initial structure for the lipid droplet was obtained by placing the set of lipid molecules (see Table 1) randomly into a three-dimensional simulation box without water or any other solvent. As CE, we used cholesterol oleate, while TG was chosen as trioleate. The system was then simulated under NpT conditions in order to reach proper density. The resulting molecular melt was next hydrated with 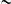
100,000 water particles and the energy was minimized, after which the system was equilibrated for 8 
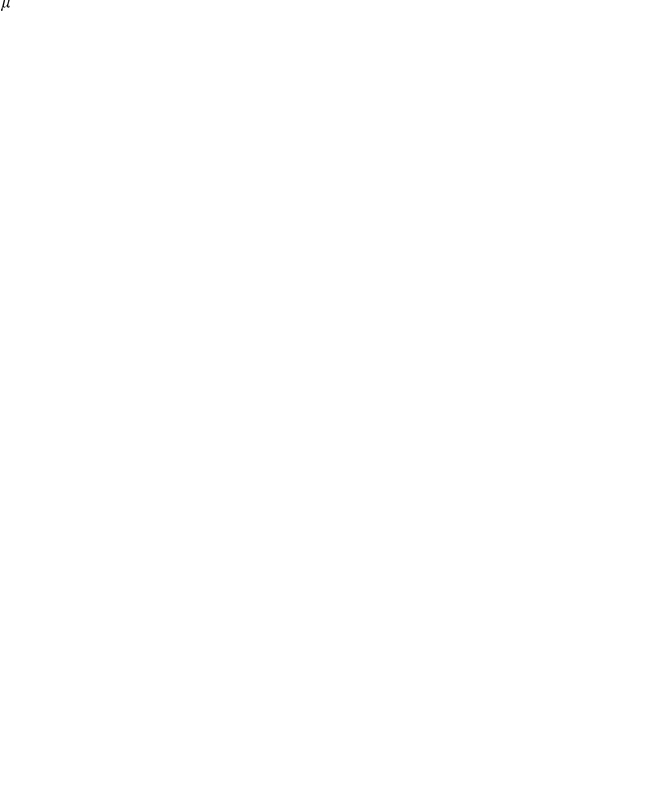
s. After equilibration, the system was simulated over a period of 4 
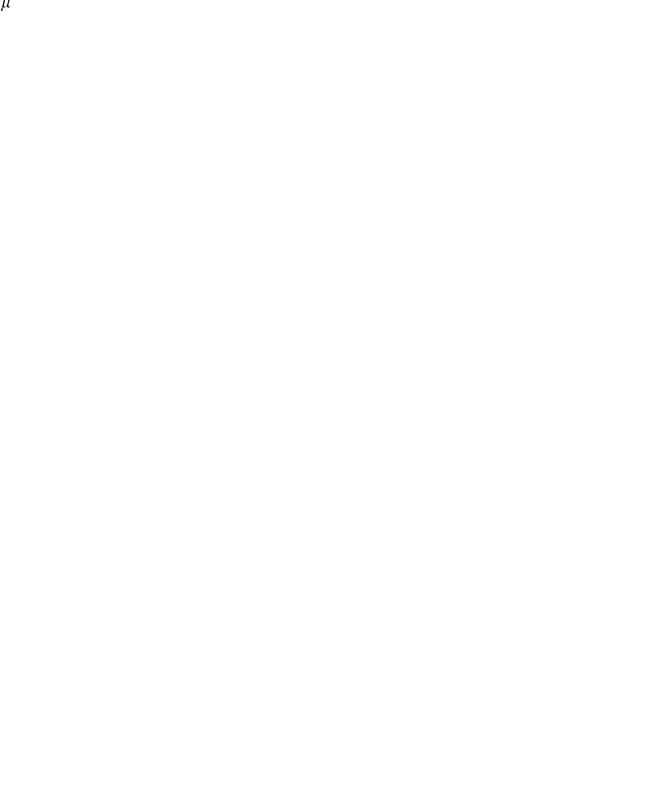
s that was used for analysis. All time scales shown here represent the realistic effective time (simulation time multiplied by the scaling factor of four) [39].

For POPC, PPC, CHOL and water, we used standard components of the coarse-grained MARTINI force field [39]. The parameters for CE and TG are those corresponding to cholesteryl-oleate and trioleate respectively, and they come from a combination of standard MARTINI-components and careful adjustments of the key particle types and angle potentials (see SI). The adjustments were justified by comparing structural properties of the molecules in bulk with extensive atomic-scale simulations [33], [34], see SI for details. Sphingomyelin (SM) in ref. [19] has been included in the POPC contribution, as SM's properties in the MARTINI description do not differ considerably from those of POPC.

Next, all-atom apoA-I molecules were generated using as a reference the molecular belt model of apoA-I for discoidal HDL [68]–[72]. The hydrophobic faces of the amphipathic helices were oriented toward the interior of the alpha helical ring but for the N-terminal part of apoA-I; the first 32 residues of the N-terminus were rotated, as in the lipid-mimetic solution NMR structure of apoA-I [22], [73], in order to have their hydrophobic face oriented towards the lipid droplet surface. These all-atom models of apoA-I were coarse grained using a pre-released version of the MARTINI force field for proteins [40] for the assignment of beads to every amino acid residue (see also ref. [36]). To obtain the initial structure of the full HDL, two coarse grained apoA-I molecules were added to the equilibrated lipid droplet (discussed above) in a double-belt conformation at a distance of 4 nm from each other.

After energy minimization, the HDL particle was subjected to very short equilibration runs using different time steps to get a stable system for a simulation with a time step of 25 fs. Finally, the particle was simulated for a total of 19 
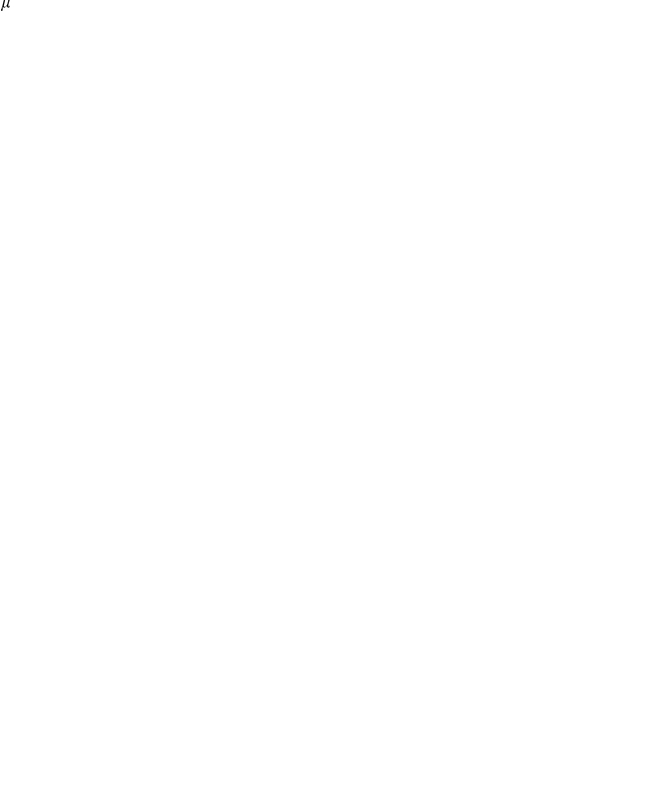
s, of which the last 4 
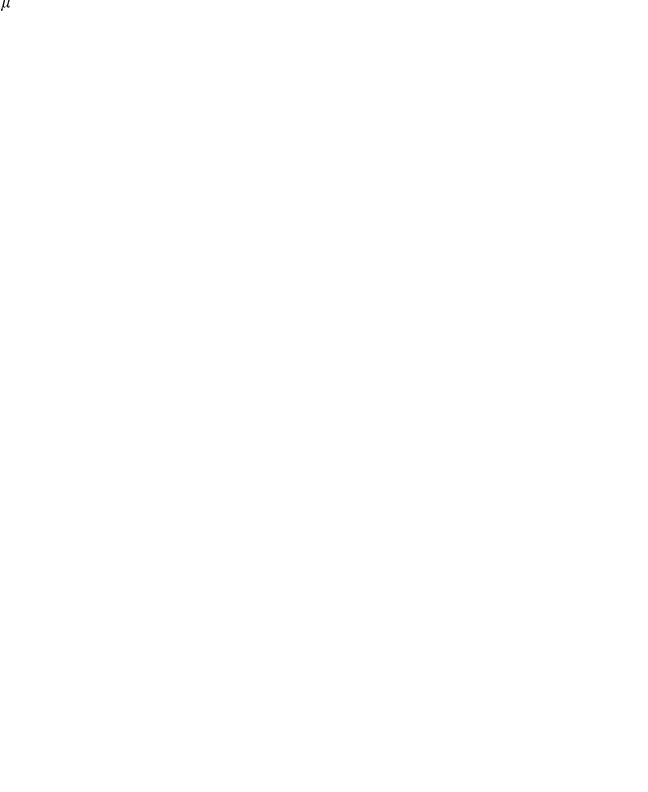
s was used for analysis.

To confirm the validity of the results, the simulations for HDL discussed in this article were complemented by several additional simulations that were started from different initial configurations. Each simulation covered a multi-microsecond time scale, and the results were found to be consistent with those discussed in this article.

The molecular dynamics simulations were performed with the GROMACS 3.3.1 package [74]. Time steps of 20 fs and 25 fs were used for integrating the equations of motion of the lipid droplet and of the full HDL, respectively. For production runs, the Nosé-Hoover thermostat [75], [76] and the Parrinello-Rahman barostat [77] were used to ensure proper NpT conditions (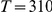 K, 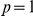 atm). Water and the lipids were coupled to separate thermostats, and the whole system was coupled to the barostat isotropically. Time constant of 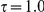 ps was used for all couplings. For non-bonded interactions, we used the standard distance of 1.2 nm [36]. The Lennard-Jones interaction was shifted smoothly to zero after 0.9 nm.

### Analysis Methods

The equilibration of the simulated lipoparticles was monitored through the numbers of intermolecular contacts between different lipid types and the radial density distributions as a function of time. The intermolecular contacts between different molecular groups were calculated using a 0.8 nm cutoff for all beads. The radial density distributions describe the number densities of the coarse-grained beads. The orientation order of CHOL and CE ring structures was measured by the order parameter 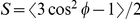, where 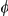 is the angle between the molecular axis and the effective normal of the lipoparticle at the location of the molecule in question. Being more specific, the molecular axis in this definition for CHOL is drawn from the beginning of CHOL (carbon in the ring of CHOL attached to the short chain) to the carbon connected to the hydroxyl group. The effective normal is the vector from the center of mass (COM) of the lipoparticle to the center of the molecular axis. For CE, an additional measure is the angle between the molecular (ring, see above) axis and the vector from the beginning to the end of the oleoyl chain.

Diffusion was analyzed by measuring the jump-length distributions of the COM positions of the lipids over a time scale 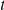. Two types of Gaussian functions were fitted to the distributions, the two-dimensional (2d):
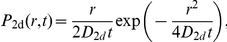
and the three-dimensional (3d):
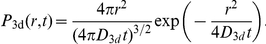



The diffusion coefficient 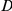 from the best fitting function (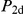 or 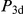) is reported, which in practice means that lipids at the water-lipid interface were found to undergo 2d diffusion, while those under the interface diffused in a 3d manner. Also, different time scales were tested and the measured 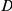 was observed to level off at long times, an indication of diffusive behavior in the hydrodynamic (long-time) limit. “Long” times here refer to times of the order of 100 ns, where 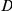 is found to level off to a well defined constant value. Examples of data for 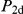 and 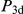 are shown in SI, including also a more detailed description of how to choose the diffusion time scale in the intermediate region under the lipid-water surface (see Text S1, and Figure S6).

In many-component systems such as the present one, the diffusion of different molecular components may take place at different rates, and it is not obvious that CG models account for this aspect correctly. For the MARTINI model used here, we have previously confirmed that this is not an issue. For instance, Niemela et al. [78] recently used atomistic and coarse-grained models to show that the protein diffusion coefficient was about 10 times smaller compared to that of lipids, and the diffusion mechanisms of lipids and proteins was similar in both models. Ramadurai et al. [79] studied the influence of membrane thickness (hydrophobic mismatch) with several peptides using both FCS measurements and coarse-grained simulations and found essentially quantitative agreement for the peptide diffusion coefficients after the MARTINI results had been scaled by a factor of 4. The simulations were also in agreement with experiments for the trend predicted with increasing hydrophobic mismatch. Further, Apajalahti et al. [61] considered the lateral diffusion of lipids in many-component protein-free membranes and found the diffusion of lipids in raft-like membrane domains (in the liquid-ordered phase) to be about 10 times slower compared to diffusion in domains that were in the liquid-disordered phase. Therefore, diffusion has been studied in several multi-component lipid systems, and the MARTINI models have been found to be consistent with experiments.

The solvent accessible surface area (SASA) of hydrophobic, hydrophilic and all-protein residues were measured using a radius of the solvent probe of 0.56 nm (the all-atom 0.14 nm radius of the solvent probe is converted to the coarse-grained one because one water bead corresponds to four water molecules) inside the GROMACS program g
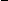
sas. The SASA values were averaged over the entire trajectory used for analysis. The root mean square fluctuations (RMSFs) of protein alpha carbons were measured for both apoA-I chains to monitor protein flexibility.

Lipid-protein interactions were monitored through the number of intermolecular contacts and their lifetimes for every lipid component with the protein. Annular lipid molecules, defined as those with any bead within 8 Å of any protein bead, were monitored over the analyzed trajectory. The average percentage of the number of contacts of each lipid component with the protein residues were estimated separately for each of the following moieties of every lipid component: POPC (polar head group, glycerol backbone, oleoyl and palmitoyl chains), PPC (polar head group, glycerol backbone and palmitoyl chain), CHOL (short acyl chain, sterol ring), CE (short acyl chain, sterol ring, and oleate chain) and TG (glycerol backbone, *sn*–1, *sn*–2, and *sn*–3 chains). Cholesterol-protein interactions were also tested by measuring the average number of contacts per protein residue of the cholesterol molecule with hydrophilic and hydrophobic protein residues. Additionally, for evaluation of CHOL-protein and CE-protein lifetimes, we accounted for cases where the distance between the molecules fluctuated around 8 Å: for an annular lipid, if its distance from apoA-I exceeded 8 Å temporarily for less than 10 frames (0.1 ns), the coupling was considered unbroken.

Here our primary interest is the lipid part of HDL, for which reason we have used the standard CG MARTINI model which does not enforce the full secondary structures in apoA-I. This computational efficient approach allows us to focus on generic issues such as the partitioning of lipids around apoA-I, as well as the influence of apoA-I on the disttributions of lipids in a droplet. By fine graining our equilibrated structures back to atomistic level, one could employ atom-scale simulations to elucidate the more detailed aspects of the system.

## Supporting Information

Figure S1 POPC angle distributions showing the orientation of the P-N vector and hydrocarbon chains with respect to the effective normal.
(0.18 MB PDF)

Figure S2 Distributions of TG conformations in different regions of HDL. Distributions are given in terms of the angle between the hydrocarbon *sn*-1 and *sn*-3 chains, and the angle between the *sn*-1 and *sn*-2 chains.
(0.63 MB PDF)

Figure S3 Conformations of cholesteryl ester and triglyceride in different parts of the droplet.
(0.24 MB PDF)

Figure S4 Angle distributions describing cholesteryl ester conformations.
(0.11 MB PDF)

Figure S5 Angle distributions characterizing conformations of triglyceride molecules.
(0.14 MB PDF)

Figure S6 Displacement distributions for CE (Panel A) and POPC (Panel B) together with the fits of P*_2d_* and P*_3d_*.
(0.19 MB PDF)

Table S1 Average percentages of different moieties of each lipid component interacting with apoA-I.
(0.01 MB PDF)

Text S1 Description of additional data (for Figures S1, S2, S3, S4, S5, S6 and Table S1), details of coarse-grained model construction, and description of diffusion analysis.
(0.05 MB PDF)
