# Supplementary material for: Role of Lipids in Spheroidal High Density Lipoproteins
Source: PLoS Comput Biol. 2010 Oct 28;6(10):e1000964. doi: 10.1371/journal.pcbi.1000964 (PMC2965744; doi:10.1371/journal.pcbi.1000964)
Supplement: Figure S1 — POPC angle distributions showing the orientation of the P-N vector and hydrocarbon chains with respect to the effective normal. (0.18 MB PDF) [file pcbi.1000964.s001.pdf]

Supporting Information: Figure S1

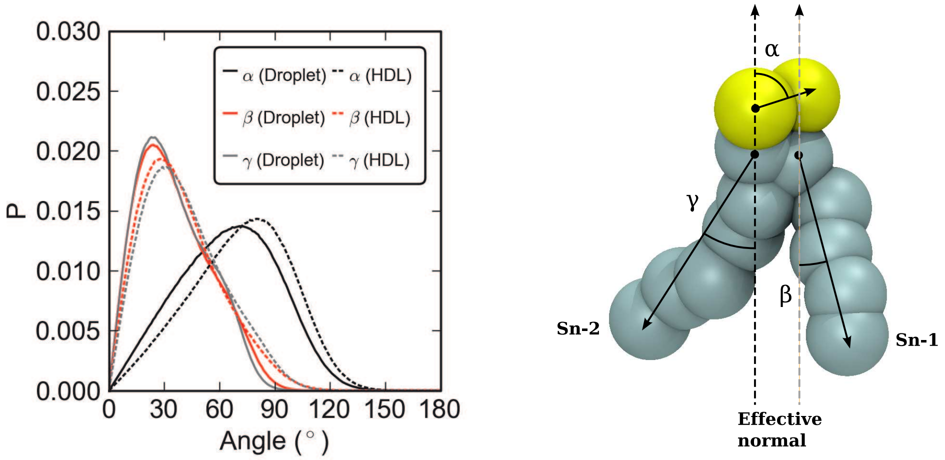

POPC angle distributions (left) showing the orientation of the P-N vector and hydrocarbon chains with respect to the effective normal. The solid lines are for the lipid droplet (without apoA-I) and the dashed lines for full HDL (with apoA-I). The angle α measures the direction of the P-N vector, and β and γ the tilt angles of the hydrocarbon *sn*-1 and *sn*–2 chains, respectively (right).
