# Supplementary material for: Role of Lipids in Spheroidal High Density Lipoproteins
Source: PLoS Comput Biol. 2010 Oct 28;6(10):e1000964. doi: 10.1371/journal.pcbi.1000964 (PMC2965744; doi:10.1371/journal.pcbi.1000964)
Supplement: Figure S2 — Distributions of TG conformations in different regions of HDL. Distributions are given in terms of the angle between the hydrocarbon sn-1 and sn-3 chains, and the angle between the sn-1 and sn-2 chains. (0.63 MB PDF) [file pcbi.1000964.s002.pdf]

Supporting Information: Figure S2

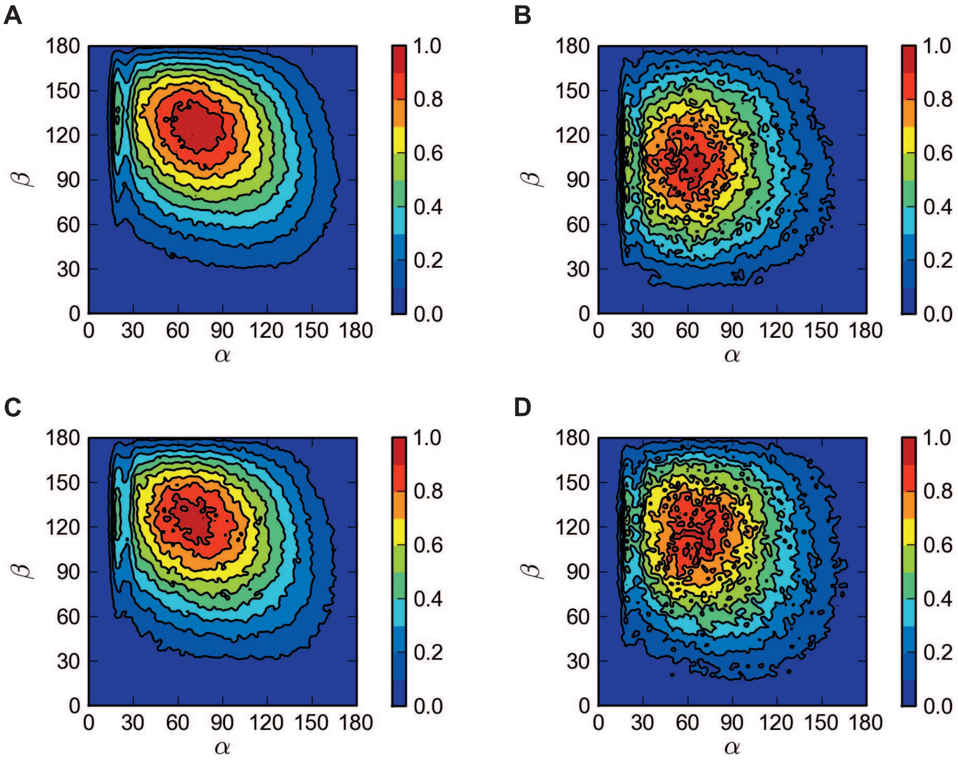

Distributions of TG conformations. The horizontal axis is the angle α between the hydrocarbon *sn*–1 and *sn*–3 chains. The vertical axis is the angle β between the *sn*–1 and *sn*–2 chains. The left panels (A,C) are for the core of the droplet ($r < 3\,\mathrm{nm}$) and the right panels (B,D) for the surface ($r > 3\,\mathrm{nm}$). The pictures at the top (A, B) are for the lipid droplet without apoA-I and those at the bottom (C, D) are for the full HDL.
