# Supplementary material for: Role of Lipids in Spheroidal High Density Lipoproteins
Source: PLoS Comput Biol. 2010 Oct 28;6(10):e1000964. doi: 10.1371/journal.pcbi.1000964 (PMC2965744; doi:10.1371/journal.pcbi.1000964)
Supplement: Figure S3 — Conformations of cholesteryl ester and triglyceride in different parts of the droplet. (0.24 MB PDF) [file pcbi.1000964.s003.pdf]

Supporting Information: Figure S3

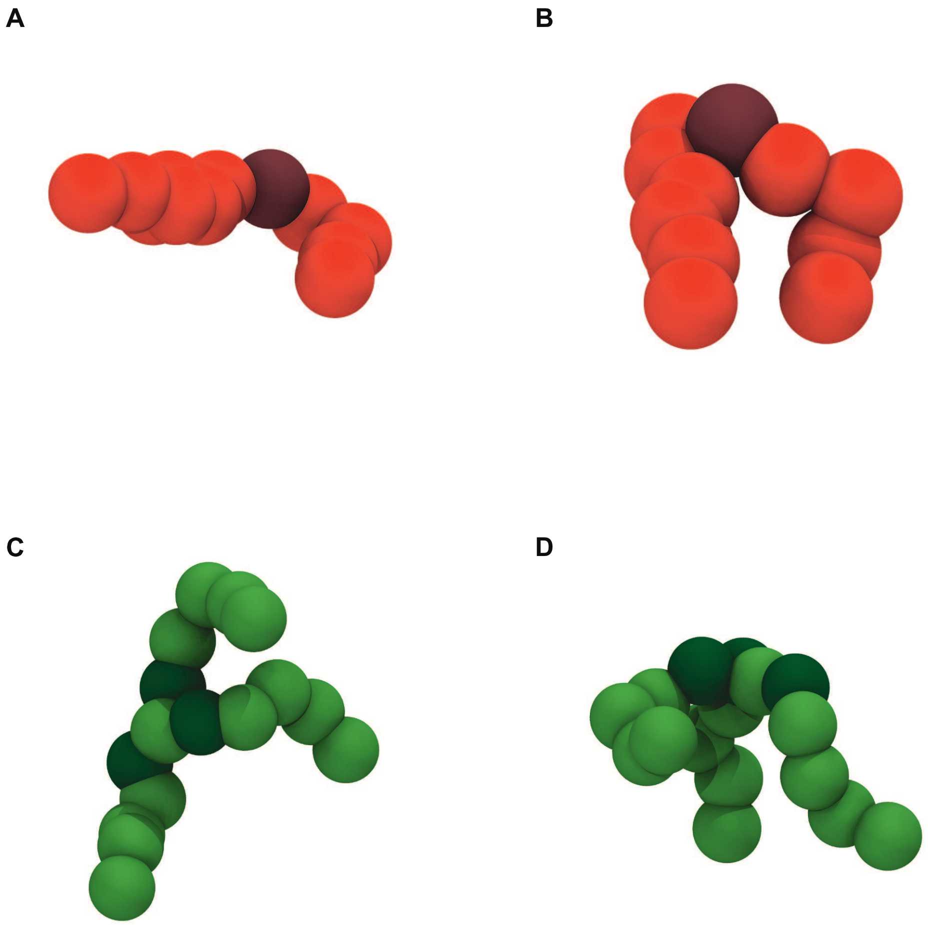

Conformations of cholesteryl ester (A,B) and triglyceride (C,D) in different parts of the droplet. Left panels (A,C) show examples of conformations in the core of the HDL particle, and right panels (B,D) show examples of conformations close to the surface. The normal of the surface points to the top of the page in each subfigure.
