# Supplementary material for: Role of Lipids in Spheroidal High Density Lipoproteins
Source: PLoS Comput Biol. 2010 Oct 28;6(10):e1000964. doi: 10.1371/journal.pcbi.1000964 (PMC2965744; doi:10.1371/journal.pcbi.1000964)
Supplement: Figure S4 — Angle distributions describing cholesteryl ester conformations. (0.11 MB PDF) [file pcbi.1000964.s004.pdf]

Supporting Information: Figure S4

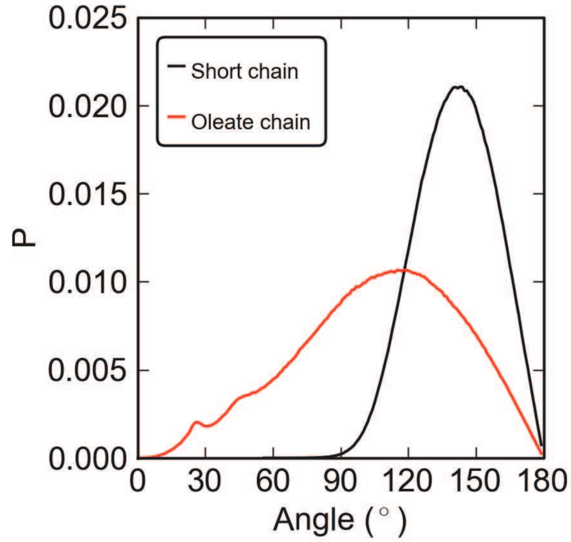

Angle distributions describing cholesteryl oleate conformations. Both the short acyl chain angle and the oleate tail angle are computed with respect to the ring structure director.
