# Supplementary material for: Role of Lipids in Spheroidal High Density Lipoproteins
Source: PLoS Comput Biol. 2010 Oct 28;6(10):e1000964. doi: 10.1371/journal.pcbi.1000964 (PMC2965744; doi:10.1371/journal.pcbi.1000964)
Supplement: Figure S5 — Angle distributions characterizing conformations of triglyceride molecules. (0.14 MB PDF) [file pcbi.1000964.s005.pdf]

Supporting Information: Figure S5

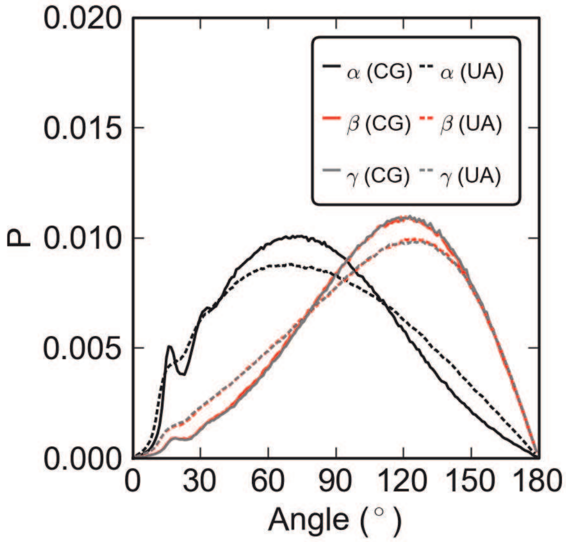

Distributions of angles characterizing conformations of triglyceride molecules. Solid lines are for the coarse-grained model and dashed lines for the atomic scale model (obtained from A. Hall et al., J Phys Chem B **112**, 13772 (2008)). The angles are defined between vectors from the central bead to the end of each chain. α corresponds to the angle between the *sn*–1 and *sn*–3 chains, β between the *sn*–2 and *sn*–3 chains, and γ between the *sn*–1 and *sn*–2 chains.
