# Supplementary material for: Role of Lipids in Spheroidal High Density Lipoproteins
Source: PLoS Comput Biol. 2010 Oct 28;6(10):e1000964. doi: 10.1371/journal.pcbi.1000964 (PMC2965744; doi:10.1371/journal.pcbi.1000964)
Supplement: Figure S6 — Displacement distributions for CE (Panel A) and POPC (Panel B) together with the fits of P2d and P3d. (0.19 MB PDF) [file pcbi.1000964.s006.pdf]

Supporting Information: Figure S6

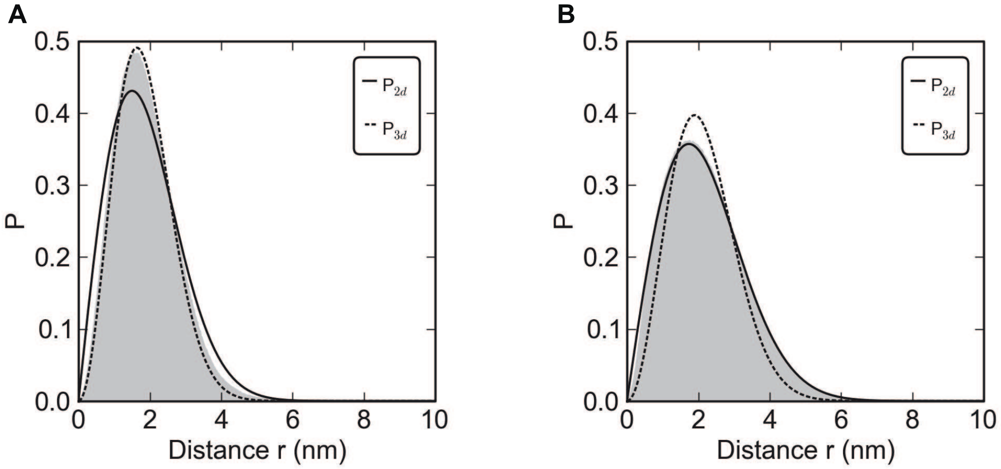

Displacement distributions for CE (Panel A) and POPC (Panel B) together with the fits of P_{2d} and P_{3d}. For CE, P_{3d} fits the data substantially better than the 2D description, while for POPC, P_{2d} describes the data significantly better compared to the 3D realization. The time interval used in the analysis was 82 ns for both distributions (A,B).
