# Supplementary material for: Role of Lipids in Spheroidal High Density Lipoproteins
Source: PLoS Comput Biol. 2010 Oct 28;6(10):e1000964. doi: 10.1371/journal.pcbi.1000964 (PMC2965744; doi:10.1371/journal.pcbi.1000964)
Supplement: Table S1 — Average percentages of different moieties of each lipid component interacting with apoA-I. (0.01 MB PDF) [file pcbi.1000964.s007.pdf]

Supporting Information: Table S1

Component	Average %
POPC polar headgroup	13.6 ± 0.1
POPC glycerol backbone	29.2 ± 0.1
POPC palmitoyl chain	25.2 ± 0.2
POPC oleoyl chain	31.9 ± 0.1
PPC polar headgroup	20.4 ± 0.8
PPC glycerol backbone	32.3 ± 0.7
PPC palmitoyl chain	47.3 ± 0.8
TG glycerol backbone	0.1 ± 14.5
TG *sn*–1 chain	24.5 ± 3.1
TG *sn*–2 chain	49.3 ± 2.8
TG *sn*–3 chain	26.1 ± 3.1
CHOL short acyl chain	9.4 ± 0.2
CHOL sterol ring	90.6 ± 0.2
CE short acyl chain	18.4 ± 0.5
CE sterol ring	65.4 ± 0.4
CE oleate chain	16.1 ± 0.6

Average percentages of different moieties of each lipid component interacting with apoA-I.
