# Supplementary material for: Role of Lipids in Spheroidal High Density Lipoproteins
Source: PLoS Comput Biol. 2010 Oct 28;6(10):e1000964. doi: 10.1371/journal.pcbi.1000964 (PMC2965744; doi:10.1371/journal.pcbi.1000964)
Supplement: Text S1 — Description of additional data (for Figures S1, S2, S3, S4, S5, S6 and Table S1), details of coarse-grained model construction, and description of diffusion analysis. (0.05 MB PDF) [file pcbi.1000964.s008.pdf]

SUPPORTING INFORMATION: Role of Lipids in Spheroidal High Density Lipoproteins

Timo Vuorela, Andrea Catte, Perttu S. Niemelä, Anette Hall, Marja T. Hyvönen, Siewert-Jan Marrink, Mikko Karttunen, Ilpo Vattulainen*

* E-mail: Corresponding author; Ilpo.Vattulainen@tut.fi

Abstract

Regarding the article in question, we provide here further details of the simulation protocol and complement the main results with additional ones.

POPC Angle Distributions

To analyze the conformations of POPC molecules we measured different angles describing the orientation of the headgroup and the hydrocarbon chains. The P-N vector (see Fig. S1) describes the orientation of the head group with respect to the effective normal. Also the tilt angles of the hydrocarbon chains were measured.

We observe that adding the protein to the lipid droplet widens the angle distributions. The effect is an indication that the protein disturbs the ordering within the surface region.

Trioleate Conformations

Figure S2 describes the internal conformations of TG molecules both in the core and on the surface of the particle. We find a change in TG conformation when it is shifted from the core to the surface. The conformation in the core is similar to the conformation in a bulk melt of TG [1]. On the surface TG adopts a more tightly packed conformation as the hydrocarbon chains get closer to each other since the polar ester bond regions seek their way to interact more favorably with water.

Lipid-Protein Interactions

Table S1 shows the average percentage of different moieties of each lipid component interacting with apoA-I residues. CHOL and CE interact mainly through the sterol ring with the protein, while POPC and PPC interact through the glycerol backbone and hydrocarbon chains, and TG through the acyl chains.

Coarse Grained Models for Cholesteryl Ester and Triglyceride

Cholesteryl ester (CE)

For cholesteryl esters, we used cholesteryl oleate, which was mapped to a coarse grained representation using 13 beads. The parameters of the model were adjusted by comparing structural properties with atomic scale simulations [2]. For that purpose we constructed a system of 1000 CE molecules, allowed it to relax to equilibrium, and then simulated the system for 900 ns to collect data for analysis.

Figure S4 shows the distributions for the orientations of both the oleate chain and the short acyl chain with respect to the ring structure. Comparing these distributions with the results from atomic

scale simulations (see Fig. 6 in Ref. [2]) shows that the coarse-grained CE model is in good agreement with the atomic scale model. In the coarse-grained model the minor peak of the oleate chain distribution has shifted about 5 degrees, while at larger angles it slightly favors extended conformations.

Typical conformations of CE are shown in Fig. S3.

We also studied the intermolecular ordering by measuring the order parameter S_{RR}, defined as

$$S_{\mathrm{RR}} = \frac{1}{2}(3\langle\cos^2\phi\rangle - 1),$$

where ϕ is the angle between the directors of two CE (or CHOL) molecules, the director describing the orientation of the ring structure along its principal axis. The results imply that the neighboring CE ring structures have a strong tendency to align themselves in the same direction, but this ordering is significant only at short distances. Comparison with the atomic scale results [2] reveals that the coarse grained model yields results in line with the atomic scale model.

Triglyceride (TG)

Trioleate molecule was mapped to a coarse-grained representation using 16 beads. The parameters in the coarse-grained model were validated by comparing its structural properties with those obtained from atomic scale simulations [1]. To this end, we constructed a system of 1000 TG molecules, pre-equilibrated it, and then simulated the model for 700 ns to collect a reasonable amount of data for analysis.

Figure S5 shows the distributions of angles describing the conformations of both coarse-grained and atomic scale models. Comparing the results for the coarse-grained and the atomic scale model shows that the coarse-grained model's behavior is in good agreement with the atomistic one.

Typical conformations of TGs are shown in Fig. S3.

In addition to analyzing intramolecular conformations, we studied intermolecular ordering using radial density distributions. According to the results, the packing of the coarse-grained triglycerides is similar to the molecules in the atomistic representation.

Diffusion Analysis

Diffusion of molecules is usually studied using mean-square displacements in the long-time limit [3]. Since diffusion in this study takes place in a confined environment, it is preferrable to use a different method. It is assumed that the centers-of-mass of molecules follow a random walk.

For simplicity, consider the one-dimensional case for a random walker. The probability that a particle, starting ar $x = 0$ at time $t = 0$, is found at time t at a distance $[x, x + \Delta x]$ from the origin can be written as [4]

$$P_{1d}(x,t)\Delta x = \frac{1}{\sqrt{4\pi D_{1d}t}} \exp\left(-\frac{x^2}{4D_{1d}t}\right)\Delta x,$$

where D_{1d} is the diffusion coefficient in one dimension.

Developing this idea further gives

$$P_{2d}(xy,t)\Delta x\Delta y = \frac{1}{4\pi D_{2d}t} \exp\left(-\frac{x^2+y^2}{4D_{2d}t}\right)\Delta x\Delta y$$

for diffusion in two dimensions, and

$$P_{3d}(xyz,t)\Delta x\Delta y\Delta z = \frac{1}{(4\pi D_{3d}t)^{3/2}} \exp\left(-\frac{x^2+y^2+z^2}{4D_{3d}t}\right)\Delta x\Delta y\Delta z$$

for three dimensions. D_{2d} and D_{3d} are diffusion coefficients for two- and three-dimensional diffusion, respectively. For spherical (and circular) symmetry with radial displacements, we consider diffusion in spherical coordinates in terms of Δr, $\Delta\theta$, and $\Delta\phi$ and their differentials. Integrating the previous equations over θ and ϕ then gives the probability distribution for finding the molecule at distance r from the origin after time t has elapsed:

$$P_{2d}(r,t)\Delta r = \frac{r}{2D_{2d}t}\exp\left(-\frac{r^2}{4D_{2d}t}\right)\Delta r$$

in two dimensions, and

$$P_{3d}(r,t)\Delta r = \frac{4\pi r^2}{(4\pi D_{3d}t)^{3/2}}\exp\left(-\frac{r^2}{4D_{3d}t}\right)\Delta r$$

in three dimensions.

The displacement distribution is a probability distribution for the distance that the center-of-mass has travelled during a given time interval. We constructed the displacement distributions for different molecule types using different time intervals (ranging from $\sim$1 ns to about 100 ns) and fitted the equations derived above to the simulation data to calculate the diffusion coefficient over the given time interval. In practice, ones gets the diffusion coefficient as a function of time t. It is expected that D levels off at long times, indicating the diffusive behavior to emerge in the hydrodynamic (long-time) limit. This is exactly what we have found in this work.

There is one point that needs to be taken into account when the long-time limit is considered. In the two-dimensional case for particles diffusing along the HDL-water interface, one can consider diffusion in the true limit of very long times. In the three-dimensional case the situation is more subtle, since the space available for three-dimensional diffusion inside HDL is confined. To avoid artifacts due to core lipids diffusing to the HDL-water interface, there is an upper limit for the time interval that one can safely use in the determination of the diffusion coefficient. A rough estimate can be made as follows. Given a diffusion coefficient of about $5 \times 10^{-8}\,\mathrm{cm}^2/\mathrm{s}$ for a molecule that is about $\ell = 1.5\,\mathrm{nm}$ from the HDL-water interface, then the diffusion time is $t_D = \ell^2/6D$. This is about 75 ns. In the same spirit, we computed the three-dimensional diffusion coefficient for core lipids over time intervals up to about $50-100$ ns and found the diffusion coefficient to converge to a fixed value well before this limit. The actual values reported in the paper were determined from the time regime where D was found to be constant.

Figure S6 shows two examples of displacement distributions together with the fitting functions. It is found that P_{2d} fits better for molecules on the surface (POPC, PPC, CHOL), and P_{3d} is appropriate for molecules in the core (CE, TG).

References

1. Hall A, Repakova J, Vattulainen I (2008) Modelling of the triglyceride rich core in lipid droplets. J Phys Chem B 112: 13772–13782.

2. Heikelä M, Vattulainen I, Hyvönen MT (2006) Atomistic simulation studies of cholesteryl oleates: Model for the core of lipoprotein particles. Biophys J 90: 2247–2257.

3. Heitjans P, Kärger J, editors (2005) Diffusion in Condensed Matter: Methods, Materials, Models. Berlin, Germany: Springer-Verlag, 2 edition.

4. Benedek GB, Villars FMH (2000) Physics With Illustrative Examples From Medicine and Biology STATISTICAL PHYSICS. New York, the USA: Springer-Verlag.
